# Beading plot: a novel graphics for ranking interventions in network evidence

**DOI:** 10.1186/s12874-024-02355-7

**Published:** 2024-10-09

**Authors:** Chiehfeng Chen, Yu-Chieh Chuang, Edwin Shih-Yen Chan, Jin-Hua Chen, Wen-Hsuan Hou, Enoch Kang, Cochrane Taiwan

**Affiliations:** 1 Cochrane Taiwan, Taipei Medical University, Taipei, Taiwan; 2 Evidence-Based Medicine Center, Wan Fang Hospital, Taipei Medical University, No. 111, Section 3, Xinglong Road, Taipei, 116 Taiwan; 3 Department of Public Health, School of Medicine, College of Medicine, Taipei Medical University, Taipei, Taiwan; 4 Division of Plastic Surgery, Department of Surgery, Wan Fang Hospital, Taipei Medical University, Taipei, Taiwan; 5 Taipei City Psychiatric Center, Taipei City Hospital, Taipei 10341, Taiwan; 6 School of Medicine, College of Medicine, Taipei Medical University, Taipei, Taiwan; 7 Cochrane Singapore, Singapore, Singapore; 8 Singapore Clinical Research Institute, Singapore, Singapore; 9 Duke-NUS Medical School, Singapore, Singapore; 10 Graduate Institute of Data Science, College of Management, Taipei Medical University, Taipei, 110 Taiwan; 11 Research Center of Biostatistics Center, College of Management, Taipei Medical University, Taipei, 110 Taiwan; 12 Biostatistics Center, Wan Fang Hospital, Taipei Medical University, Taipei, 116 Taiwan; 13 Department of Physical Medicine and Rehabilitation, Taipei Medical University Hospital, Taipei, Taiwan; 14 Department of Physical Medicine and Rehabilitation, School of Medicine, College of Medicine, Taipei Medical University, Taipei, Taiwan; 15 Institute of Health Policy & Management, College of Public Health, National Taiwan University, Taipei, Taiwan; 16 School of Gerontology and Long-Term Care, College of Nursing, Taipei Medical University, Taipei, Taiwan

**Keywords:** Network meta-analysis, Statistic plot, Decision-making, Treatment ranking

## Abstract

**Background:**

Network meta-analysis is developed to compare all available treatments; therefore it enriches evidence for clinical decision-making, offering insights into treatment effectiveness and safety when faced with multiple options. However, the complexity and numerous treatment comparisons in network meta-analysis can challenge healthcare providers and patients. The purpose of this study aimed to introduce a graphic design to present complex rankings of multiple interventions comprehensively.

**Methods:**

Our team members developed a “beading plot” to summary probability of achieving the best treatment (P-best) and global metrics including surface under the cumulative ranking curve (SUCRA) and P-score. Implemented via the “rankinma” R package, this tool summarizes rankings across diverse outcomes in network meta-analyses, and the package received an official release on the Comprehensive R Archive Network (CRAN). It includes the `PlotBead()` function for generating beading plots, which represent treatment rankings among various outcomes.

**Results:**

Beading plot has been designed based on number line plot, which effectively displays collective metrics for each treatment across various outcomes. Order on the -axis is derived from ranking metrics like P-best, SUCRA, and P-score. Continuous lines represent outcomes, and color-coded beads signify treatments.

**Conclusion:**

The beading plot is a valuable graphic that intuitively displays treatment rankings across diverse outcomes, enhancing reader-friendliness and aiding decision-making in complex network evidence scenarios. While empowering clinicians and patients to identify optimal treatments, it should be used cautiously, alongside an assessment of the overall evidence certainty.

**Supplementary Information:**

The online version contains supplementary material available at 10.1186/s12874-024-02355-7.

## Introduction

Network meta-analysis has emerged as a pivotal method to surmount the limitations inherent in the traditional meta-analysis for direct comparison of two treatments [1, 2]. This approach facilitates pairwise comparisons between all available treatments, transcending the need for direct evidence through a network model that integrates both direct and indirect effects [3–5]. In clinical scenarios spanning diverse specialties, clinicians often confront the task of choosing from multiple alternative treatments. Network meta-analysis addresses this complexity by furnishing comprehensive insights into the effectiveness and safety of various treatment options. Consequently, this method has garnered widespread acceptance and endorsement through articles that have introduced it to clinicians [2, 6–8]. The World Health Organization has also demonstrated support for development of the method and has established guidelines based on the outcomes of network meta-analysis [9]. However, healthcare providers and patients might encounter challenges stemming from the intricate nature of network meta-analysis and the multitude of treatment comparisons [10]. Consequently, the post-network meta-analysis treatment ranking emerges as a valuable technique for aiding decision-making, benefiting both healthcare providers and patients alike [11–15].

Navigating decisions based on numerous effect sizes accompanied by confidence intervals across various outcomes with multiple pairwise comparisons is a daunting task. To address this complexity, certain metrics have been introduced to offer clinicians and patients concise summaries of network evidence [13, 14, 16]. These treatment ranking metrics predominantly rely on probabilities, encompassing probabilities of treatments for each conceivable rank, the probability of achieving the best treatment (P-best), surface under the cumulative ranking curve (SUCRA), and the P-score [13, 14, 16]. The notion of probabilities assigned to treatments for each potential rank serves as not only a metric for treatment ranking but also as the foundational framework for other ranking metrics. Yet, it presents an abundance of information spanning various ranks and treatments. Essentially, these probabilities form a matrix, providing intricate data rather than straightforward metrics. P-best, derived from the probability matrix, allows clinicians and patients to focus on the potential best treatment option within the spectrum of available choices, serving as an intuitive ranking metric. SUCRA encompasses comprehensive treatment ranking metrics by incorporating probabilities from each conceivable rank through Bayesian simulation, while P-score is SUCRA-like metrics calculated by frequentist approach. Although SUCRA and P-score calculations are intricate, their outcomes offer simplified insights for clinicians and patients. This comprehensive insight aids stakeholders in grasping the complex nuances behind the expansive results of network evidence [12, 15]. Numerous network meta-analyses have embraced and reported these treatment ranking metrics for clinical suggestions or recommendations [17–19].

The demonstration of intervention comparisons via well-crafted graphics holds paramount importance in the integration of network meta-analysis into the decision-making process. Numerous graphic tools tailored for enhancing decision-making have emerged, designed to foster effective communication between healthcare providers and patients [14, 20–22]. Constrained by the intricacies of visualization, we believe that a visually intuitive graphic can enhance the comprehensibility of intricate concepts and subsequently mitigate the challenges associated with decision-making resulting from the misinterpretation of clinical interventions in the context of network meta-analysis application. With this perspective, our objective was to introduce a graphic design capable of not only presenting intricate rankings of multiple interventions in a comprehensive manner, but also alleviating the constraints posed by varying levels of familiarity with network meta-analysis in the decision-making process.

## Methods

The innovative visual tool known as the “beading plot” was initially conceptualized by a team from Cochrane Taiwan. This article serves as a comprehensive introduction and demonstration of this novel graphical representation. Through multiple rounds of discourse and iterative testing conducted during regular team meetings, initial concepts were translated into practical implementation using the *R* package “*rankinma*” [23]. This package was developed to encompass the summary of rankings across diverse outcomes within network meta-analyses. Its functionalities encompass aggregating treatment ranking metrics from network meta-analysis results and generating inventive graphics to visualize these metrics. Notably, the package incorporates a function titled `PlotBead()` which facilitates the creation of beading plots. This function operates on well-structured data frames comprising three columns: treatment, ranking metrics, and outcome. The Comprehensive R Archive Network (CRAN) conducted a thorough examination and assessment of the package, followed by an official release on their platform. Accompanying this release is a comprehensive package manual [23].

### Computation and common plots of ranking metrics

As mentioned above, P-best, SUCRA, and P-score are commonly used treatment ranking metrics, and their calculation rules or formulas are as follows [14]. Firstly, a straightforward method for obtaining probabilities for the distribution of parameters has been proposed in the Bayesian approach; wherefore P-best can be derived using relevant method. Specifically, each treatment *i* is prioritized in each Markov chain Monte Carlo (MCMC) cycle based on the expected effect size. The proportion (i.e. P(*i* = 1)) that a certain therapy ranks first is the best among the available treatment alternatives is provided by the percentage of cycles in which it ranks first out of all the others. For ranking second best, third best, and so forth until the last possible rank, equivalent probabilities are calculated (i.e. P(*i* = *b*), *b* = 1, ., *l*). Italic character *b* denotes possible rank, and *l* denotes the last possible rank. Probability of each treatment add up to one, and similarly sum of probability of each rank also equal to one.

Cumulative probability for each treatment (P_*cum*_(*i*, *b*), *b* = 1, …, *l*) is derivable from the abovementioned information, and can be plotted by line chart. Then, the surface under the cumulative probabilities on the line chart can be further computed as a global metrics of treatment ranking. The global metrics in terms of SUCRA for each treatment is calculated using the following formula:

$$SUCR{A_i} = \frac{{\Sigma _{b = 1}^{l - 1}{P_{cum}}(i,b)}}{{l - 1}}$$

Probabilities of treatment ranking and are commonly illustrated by a series of line charts or stacked bar chart, and the cumulative probabilities are usually displayed by either series of line chart or a multi-line chart [14, 24–26]. With regard to global metrics of treatment ranking, heat plot is a classic graphics for depicting both SUCRA and P-score on a specific outcome, and can be carried out using package *netmeta* in *R* [27]. The rank-heat plot serves as an alternative for summarizing findings from network meta-analysis and was well-designed in 2016 [28]. Spie chart provides another option for showing multiple SUCRA values of outcomes for a specific treatment, and it is also proposed to be illustrated using *R in 2020* [11].

## Design of beading plot

**Fig. 1:**
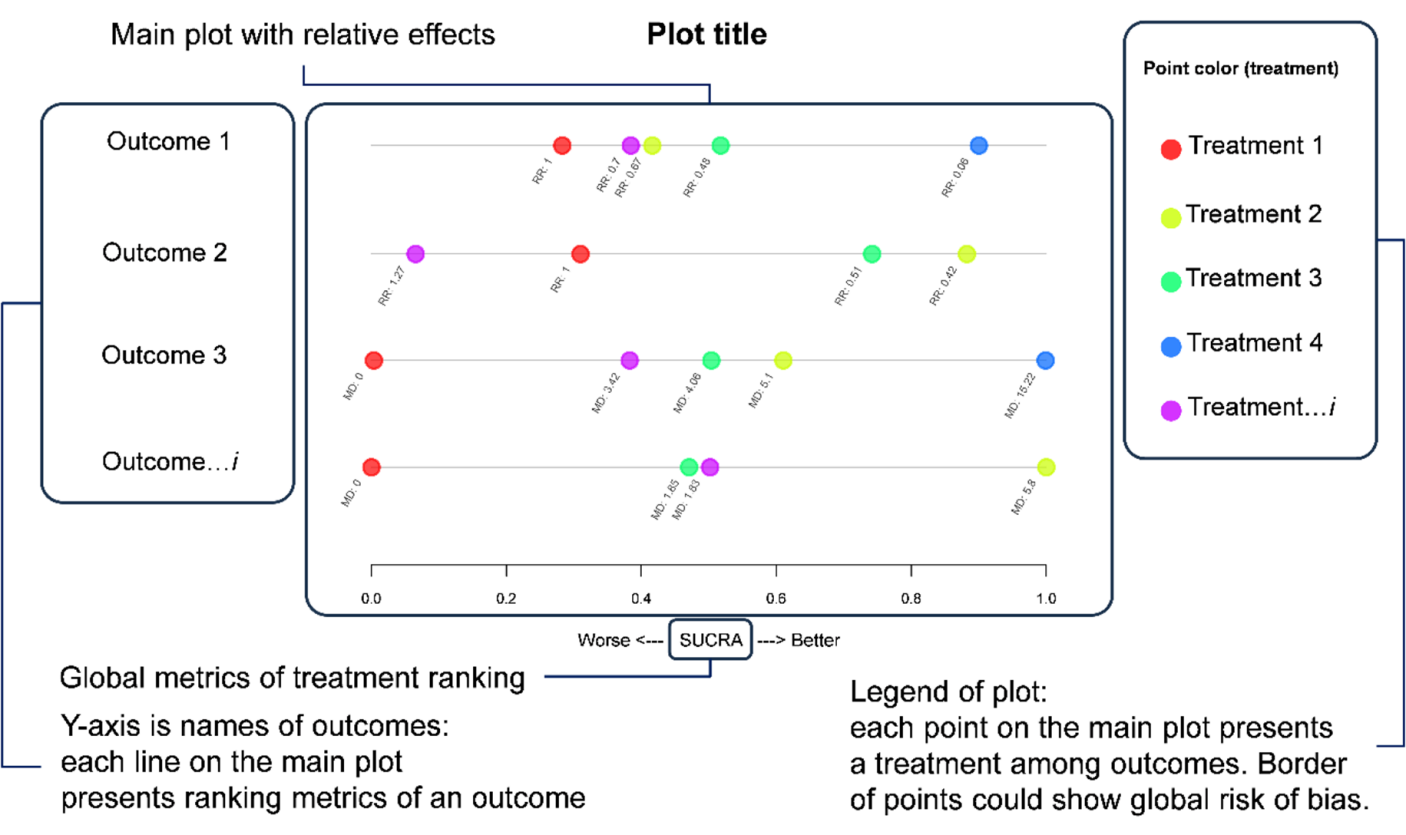
Graphical explanation of beading plot

In order to present a comprehensive overview of multiple outcomes from network evidence synthesis, we introduced the beading plot, an adaptation of the number line plot. This novel visualization method showcases the collective metrics for each treatment across various outcomes of interest. The beading plot employs a range of 0 to 1 to effectively represent global metrics, encompassing both SUCRA and P-score measurements. Furthermore, it facilitates the portrayal of not only the summary rank probabilities for each treatment but also the estimate of a treatment being the best in terms of P-best. Notably, the lines on the beading plot span the continuous interval between 0 and 1, rather than discrete integers, with each line signifying a distinct outcome. Colored beads corresponding to treatments embellish the plot, accompanied by labels that elucidate their relative effects. Border of the plot could be used for showing risk of bias. This innovative visualization technique, aptly termed the “beading plot,” is presented in Fig. 1 to elucidate its structure and functionality.

To present a comprehensive overview of multiple outcomes in network evidence synthesis, we developed the beading plot, inspired by the number line plot, to visualize the overall metrics of each treatment across various outcomes. The beading plot employs a scale ranging from 0 to 1 to accommodate global metrics, encompassing SUCRA and P-score. In addition to the summary values of rank probabilities of every treatment, the beading plot can also be applied to illustrate the probability of been the best treatment in terms of P-best. Notably, the beading plot features continuous lines within the 0 to 1 range, each representing a distinct outcome. Treatments are color-coded as indicated in the legend. This innovative visualization technique, referred to as the “beading plot,” provides a vivid representation, as depicted in Fig. 1.

## Dataset for demonstration

As illustrative instances of treatment ranking plots, including the beading plot, this study employed a dataset derived from a network meta-analysis conducted by Kang et al. (2022) [29], which investigated the effects and safety of lumbar fusion techniques in patients with lumbar spondylolisthesis. The meta-analysis encompassed 15 randomized controlled trials, involving a collective sample size of 992 cases. The reported outcomes were fusion rate, Oswestry Disability Index (ODI) score, adverse event, and operative time.

## Software

Pooled analysis was conducted in the random-effects model using the contrast-based method in terms of frequentist approach. Network meta-analysis was carried out by function `netmeta()` in package *netmeta* (version 2.8-2). The ranking plots were further illustrated by authors based on the extracted metrics since package *netmeta* was designed to conduct a well network meta-analysis using frequentist methods but with limited graphics for treatment ranking [27]. We used *R* to carry out the network meta-analysis, and produced various graphics of commonly used treatment ranking plots including rank probabilities plots (i.e. bar chart and line chart), cumulative probability plots (both bar chart and line chart), heat plot, as well as spie plot using R package *rankinma* (version 0.2.2). The rank-heat plot was executed utilizing the platform offered by the Knowledge Translation Program in Canada. The beading plot, on the other hand, was generated using the R package *rankinma*, and the procedural steps along with the corresponding R code for producing the beading plot are detailed in File S1. Relevant information was presented in Table S1.

## Results

Based on the available dataset from the synthesis by Kang et al. (2022), there were six treatments, including circumferential fusion, minimally invasive transforaminal interbody fusion (MTLIF), posterolateral fusion(PLF), posterior lumbar interbody fusion (PLIF), transforaminal interbody fusion (TLIF), and extreme lateral interbody fusion (XLIF). Six-node network model was formed for fusion rate and adverse event, while ODI can be analyzed based on a four-node consistency model, encompassing MTLIF, PLF, PLIF, and TLIF. Result of operative time was reported for five surgical interventions, including MTLIF, PLF, PLIF, TLIF, and XLIF. The reproduced results in the present study were similar to the results in the study by Kang et al. (2022) [29], and reproduced results were reported in Figure S1. Table 1 presented further generated information including probability of being the best (P-best), SUCRA value, and P-score.

**Table 1:** Finding summary table

Outcome / Treatment	*P*-best	SUCRA	*P*-score
Fusion rate			
Circumferential	0.999	1.000	0.999
MTLIF	0.000	0.363	0.336
PLF	0.000	0.264	0.252
PLIF	0.000	0.443	0.460
TLIF	0.000	0.359	0.357
XLIF	0.001	0.571	0.596
**Oswestry Disability Index**			
MTLIF	0.327	0.366	0.327
PLF	0.714	0.738	0.714
PLIF	0.660	0.583	0.660
TLIF	0.299	0.312	0.299
**Any adverse event**			
Circumferential	0.120	0.505	0.548
MTLIF	0.594	0.823	0.837
PLF	0.061	0.500	0.536
PLIF	0.027	0.361	0.307
TLIF	0.026	0.377	0.375
XLIF	0.172	0.434	0.396
**Operative time**			
MTLIF	0.058	0.298	0.306
PLF	0.500	0.771	0.804
PLIF	0.091	0.423	0.363
TLIF	0.114	0.519	0.507
XLIF	0.237	0.490	0.520

MTLIF, minimally invasive transforaminal interbody fusion; P-best: probability of achieving the best treatment; PLF, posterolateral fusion; PLIF, posterior lumbar interbody fusion; SUCRA: surface under the cumulative ranking curve; TLIF, transforaminal interbody fusion; XLIF, extreme lateral interbody fusion

### Commonly used treatment ranking plots

Before the beading plot is proposed, several basic plots can be used for displaying probability of every surgical procedure on each possible rank in the analysis of fusion rate. For instance, the probabilities can be illustrated on a series of line charts (Figure S2) or on a multi-line plot (Figure S3). The plot indicated the most possible rank for each lumbar fusion procedure by the highest peak in a single line chart for the specific module. Similarly, the same information can be concisely plotted on a bar chart of cumulative probabilities (Figure S4), on which the most possible rank for each lumbar fusion procedure can be identified with the highest bar across multiple probability bars of ranks. Furthermore, the possibly optimal lumbar fusion procedure for achieving fusion can be illustrated by a series of cumulative line charts (Figure S5) or on a multi-line plot (Figure S6). A possible top one choice could be the surgery with the earliest achievement of 100% in cumulative probability.

Although abovementioned plots can exhibit details on the probabilities of each treatment in every possible rank, patterns in the plots sometimes were unclear due to increased numbers of treatments without any global metrics in terms of SUCRA or P-score. Simple bar chart and heat plot could be applied to the visualization of the global metrics on the treatment ranking amongst the lumbar fusion techniques in the outcome of fusion rate (Figure S7 and S8). The two plots placed the optimal choice of lumbar fusion technique in the left side followed by the second choice, the third choice, and till the last one. Global metrics of treatment ranking can also be displayed by heat plot (Figure S9).

Nevertheless, all the graphics above only displayed the outcome of fusion rate, but probabilities or global metrics (i.e. SUCRA or P-score) for the other outcomes ought to be drawn in other plots (Figure S10 to S33). Besides, all the global metrics of the four outcomes can be integrated in spie plot by lumbar fusion procedures (Figure S34). The rank-heat plot is a good graphic design to summarize findings of all outcomes across all treatments in a network meta-analysis (Figure S35). The spie plots were clear graphics to show the within-treatment comparison of outcomes. Scatter plot (Figure S36) was also a common graphics for integrated information of treatment rankings and outcomes, while they can only contain two outcomes at once.

## Beading plot

**Fig. 2:**
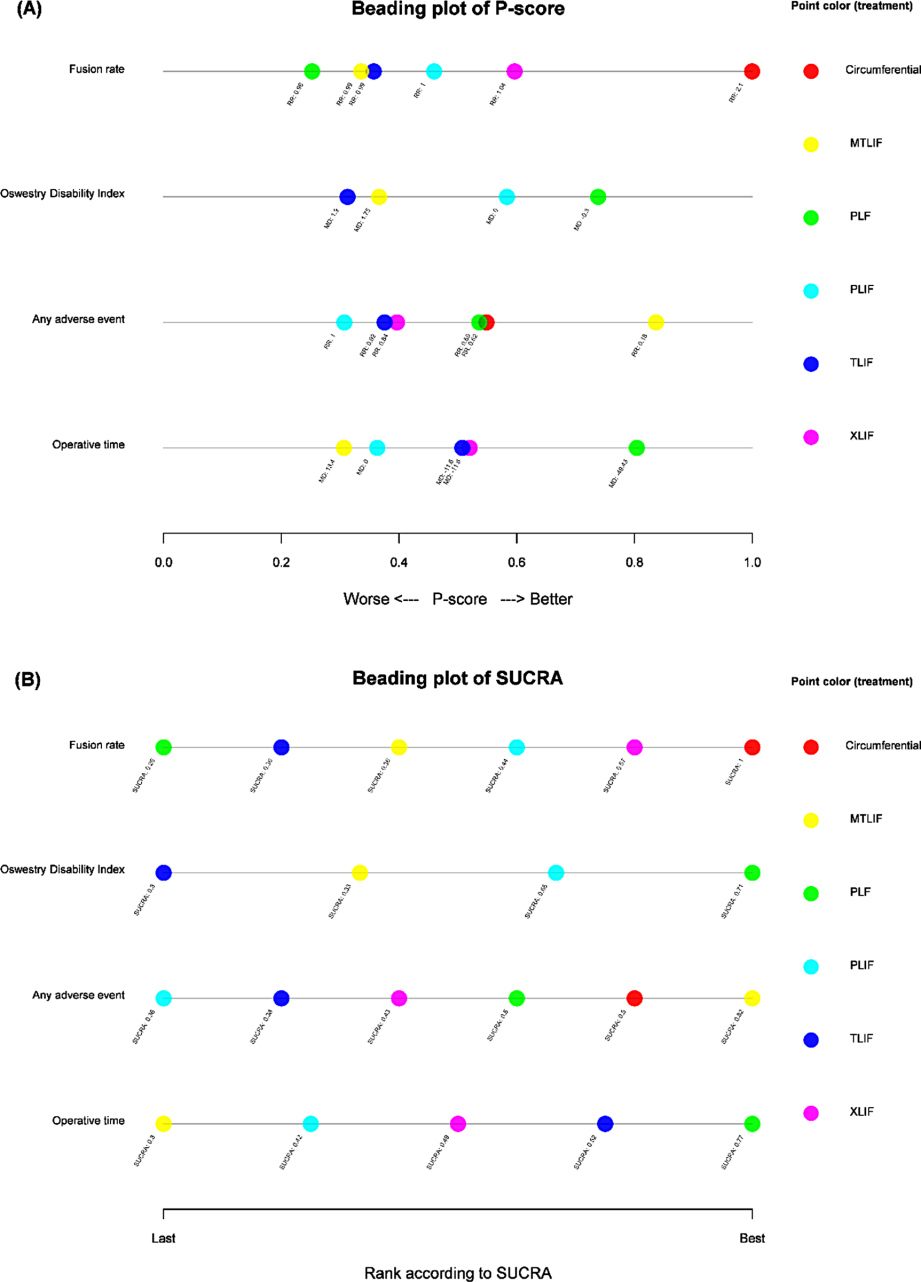
Beading plot for (**A**) P-score and (**B**) SUCRA of exercise-based treatment modules on four outcomes. MTLIF, minimally invasive transforaminal interbody fusion; PLF, posterolateral fusion; PLIF, posterior lumbar interbody fusion; TLIF, transforaminal interbody fusion; XLIF, extreme lateral interbody fusion

Figure 2 summarized the four outcomes of interest amongst the six lumbar fusion procedures. As a probability line plot, the x axis of the beading plot ranged from 0 to 1 for either P-score (Fig. 2A) or SUCRA value (Fig. 2B) or of all possible procedures in consistency model, and Figure S37 showed the plot using color-blind-friendly schemes. Due to the four outcomes, the y axis of the beading plot had four levels. P-score and SUCRA value had been colored by groups of lumbar fusion techniques. Each group had constantly been labeled across the four outcomes using the same color. It is obvious to identify the circumferential fusion might be a better strategy for fusion rate without increased risk of adverse event. On the other hand, TLIF or MTLIF might be not the first recommended procedures in managing lumbar spondylolisthesis.

## Discussion

This article presents an exposition of various frequently employed treatment ranking plots, along with an elaborate exploration of the novel graphical representation known as the beading plot. The beading plot serves to summarize global metrics (such as P-best, SUCRA, or P-score) pertaining to each outcome with treatments. The principal focus of this article centers on the introduction of the beading plot, supplemented by insightful discussions elucidating its distinctive attributes, in conjunction with other prevalent treatment ranking plots. While the other plots are not exhaustively elaborated within this present work due to their prior coverage in existing references [14, 24–26], pertinent information is systematically presented in tabular form to afford a lucid comprehension of the characteristics underpinning the beading plot, in tandem with commonly utilized treatment ranking plots. This approach is adopted as there appears to be a dearth of comprehensive literature consolidating the graphical depictions for a holistic grasp of the subject matter, through the enumeration of inherent traits, advantages, and limitations across the array of plots. A summary of these aspects is collated in Table 2.

**Table 2:** Features of graphics for metrics of treatment ranking

Feature	Simple bar chart	Stacked bar chart	Simple line chart	Multi-line chart	Scatter plot	Heat plot	Rank-heat plot	Spie plot	Beading plot
Probabilities of ranks	✓	✓	✓	✓					P-best
Global metrics	✓		✓	✓	✓	✓	✓	✓	✓
Numbers of outcomes	1	1	1	1	2	1	Multiple	Multiple	Multiple
Numbers of treatments	1	1	1	1	Multiple	1	Multiple	1	Multiple
Direction dependency	Moderate	Moderate	Moderate	Moderate	High	Low	Low	Low	High
Color dependency	Low	High	Low	High	Moderate	High	Moderate	Moderate	High
Text dependency	Low	Low	Low	Low	Moderate	Low	Moderate	Moderate	Low
Intuitive visualization	High	Moderate	High	Moderate	Moderate	High	Moderate	Moderate	High
Information richness	Low	Moderate	Low	Moderate	Moderate	Low	High	High	Moderate
Extensibility of outcomes	Low	Low	Low	Low	Low	Moderate	Moderate	Moderate	High
Extensibility of treatments	High	Moderate	High	Moderate	Moderate	High	Moderate	Low	High
Ease of producing	High	Moderate	High	Moderate	Low	High	High	Low	High
Software	ADDIS, R, SAS, STATA	ADDIS or STATA	ADDIS, R, SAS, STATA	R or STATA	ADDIS, NMAStudio, STATA,	CINeMA or NMAStudio	R or Rank-Heat Plot	R	R or RankINMA

P-best: probability of achieving the best treatment

As the beading plot is placed with other commonly used treatment ranking graphics, it is obvious to find the unique style with sufficient information but simple design in the newly developed plot even without details of probabilities of every treatment on each possible rank because it displays the global metrics on the treatment ranking differently amongst the treatment ranking plots. Indeed, some of the other treatment ranking graphics illustrate probabilities of treatment on each possible rank, but global metrics for treatment ranking are still the useful indexes for choosing the best treatment in network evidence for clinical practice [12, 14]. During the process of simplifying information, it is imperative to acknowledge that global metrics may inadvertently sacrifice the granularity of ranking probabilities, a facet that can hold significance under certain circumstances. Therefore, a thorough comprehension of the evidence, juxtaposed with a variety of metrics, remains paramount for clinicians and researchers who are interpreting beading plots.

The beading plot has been designed to demonstrate global metrics that are also widely used in other well-known plots such as bar chart for SUCRA value or heat plot for P-score [13, 14, 24–27]. In other words, information delivered by the beading plot can be compatible with the other commonly used treatment ranking graphics. However, the bar chart and the heat plot are designed for single outcome, and the default of the both plots is to display treatments according to the global metrics in descending order. In contrast with the two graphics, the beading plot breaks the limitation of single outcome, and can be easily extended to several outcomes. The extension succeeds to parallelize multiple outcomes of interest in a single plot. Actually, this idea is inspired by the multidimensional scaling approach of treatment ranking in terms of scatter plot, rank-heat plot, and spie chart for SUCRA values [11, 24, 28]. Unfortunately, a scatter plot is commonly designed for two dimensions, in which only two outcomes can be put together [24].

Concerning rank-heat plot, it emerges as a compelling visual tool for effectively synthesizing the intricate findings of network meta-analysis [28]. While the circle-shaped design, embellished with a captivating color gradient, adds to its aesthetic appeal and fosters a sense of balance among treatments and outcomes, it concurrently obscures the intuitive perception of the rank order. Moreover, the proliferation of treatments or outcomes exacerbates the mutual compression of available space within the confines of this circular representation. As the plot becomes increasingly crowded with outcomes or treatments in a limited circle, decision-making may pose a greater challenge due to the diminished intuitive perception. In pursuit of heightened extensibility and clarity, an alternative design approach, characterized by a simpler and more adaptable horizontal or vertical layout, presents itself as a potential solution. Therefore, the beading plot has been designed based on number lines, promoting a smoother assimilation of information and enhancing extensibility to accommodate more treatments and outcomes.

With regard to the spie chart, it can display various outcomes on a single plot [11], but it is a treatment-based ranking plot. The spie chart is designed to plot treatment by treatment although a single spie chart has no limitations on the numbers of outcomes. Accordingly, researchers or clinicians still need several spie charts or a series of spie charts to complete the information on treatment ranking amongst multiple outcomes. Taking features of the scatter plot and spie chart into consideration, the beading plot has been successfully designed to provide a concise summary of treatment ranking on various outcomes in a single plot.

### Implication of beading plot

As previous mentioned, network meta-analysis provides results of multiple-treatments comparisons and enables ranking among interventions, which also renders the presentation of results through understandable graphics challenging. By clearly ranged several interventions on a line from most to least probabilities, no matter with different metrics, beading plot makes the ranking of competing interventions clear on glance, which could not only decrease the difficulty of understanding complex results of a network meta-analysis and help researcher cross the learning threshold, promoting more academic exchange, but also lower the barriers of implication of network meta-analyses on decision-making, being more applicable in clinical settings for better demonstrating multiple interventions to patients by health providers. An online web for illustrating beading plot (https://rankinma.shinyapps.io/RankINMA), that is named RankINMA, has been well-established by our methodology team, and it would make beading plot being available to researchers, health providers, and patients without programming skills.

### Limitation

At least three issues ought to be aware before the application of the beading plot in clinical practice although it owns several advantages as mentioned above. Firstly, the beading plot is just a way to display multiple global metrics for choosing the optimal treatments, and the global metrics are the key for clinical decision. The beading plot cannot determine which treatment rank metric is the best although this article raises examples of the beading plot by using three commonly used global metrics in terms of SUCRA value, P-score, and P-best. The suitability of utilizing the beading plot hinges upon selecting the appropriate global metric for treatment ranking in alignment with the treatment hierarchy question [30]. If clinicians or researchers seek to underscore the treatment most likely to exhibit the most favorable mean value concerning the studied outcome, P-best emerges as an appropriate choice. In contrast, if the objective is to ascertain which treatment surpasses the largest fraction of competitors, SUCRA or P-score may prove more appropriate. Given the distinct inquiries addressed by these metrics, it is advisable to present the relative effects or probabilities of each treatment across all conceivable ranks in conjunction with the beading plot. The Litmus Rank-O-Gram provides a useful model for integrating various metrics into one visualization [21]. Secondly, the beading plot does not guarantee the certainty of the synthesis, and the interpretations of the beading plot are necessary to take certainty of evidence into consideration. For an example, poor network structure could diminish certainty and confidence in the synthesis result. Hence, it would be beneficial to present the network structure alongside global metrics of treatment ranking, such as network overlays or nested radial SUCRA plots [21]. For another, global metrics may face potential influence due to incomplete outcome reporting in certain studies, warranting cautious interpretation of the results. In cases where there is incomplete outcome reporting and limited information, a viable solution is to convert or estimate data based on available information using DECoMA [31], particularly when researchers are unable to contact corresponding authors to obtain desired statistics. Similarly, the incomplete network across multiple outcomes would threaten the appropriateness of the use of the beading plot. In other words, clinicians, researchers, and patients should be very careful if the beading plot is based on the evidence with low certainty or incomplete network. Besides, how to incorporate certainty in the beading plot is worthy of further studies. It would be advisable to display various information simultaneously using different visualizations. Thirdly, treatments in the beading plot are labeled by colors, while they may not easily be recognized when network evidence consists of a ton of treatments. Keeping top five ranks on the beading plot may be a solution for simplifying the huge information. Despite its aforementioned limitations, the beading plot remains somewhat useful for researchers and clinicians seeking an overview of network evidence, as evidenced by its application in oncology and gastroenterology studies following the release of the R package rankinma on CRAN [32, 33].

## Conclusions

The beading plot is a valid and viable graphics which visualizes treatment ranking of multiple outcomes simultaneously and intuitively. It can be used for drawing various global metrics of treatment ranking such as SUCRA, P-score, and P-best. The probability-like number lines might be a reader-friendly graphics, and would support decision-making by summarizing abundant information from network evidence. By providing an overview with appropriate displayed information, the beading plot may have the potential to empower clinicians and even patients to identify optimal treatments from complicated comparisons and multiple outcomes. However, the beading plot should not be used without the consideration of certainty of the entire evidence.

## Electronic supplementary material

Below is the link to the electronic supplementary material.

Supplementary Material 1

## Data Availability

The datasets generated during and/or analysed during the current study are available from the corresponding author on reasonable request.

## Abbreviations

CRAN: Comprehensive R Archive Network
MCMC: Markov chain Monte Carlo
MTLIF: Minimally invasive transforaminal interbody fusion
P-best: Probability of achieving the best treatment
PLF: Posterolateral fusion
PLIF: Posterior lumbar interbody fusion
SUCRA: Surface under the cumulative ranking curve
TLIF: Transforaminal interbody fusion
XLIF: Extreme lateral interbody fusion
