# Supplementary material for: Beading plot: a novel graphics for ranking interventions in network evidence
Source: BMC Med Res Methodol. 2024 Oct 9;24:235. doi: 10.1186/s12874-024-02355-7 (PMC11462909; doi:10.1186/s12874-024-02355-7)
Supplement: Supplementary file 1 — Supplementary Material 1 [file 12874_2024_2355_MOESM1_ESM.pdf]

# SUPPLEMENTARY FILE

## Beading plot: A novel graphics for ranking interventions in network evidence

Running title: Beading plot of treatment ranking

**Chiehfeng Chen**, M.D., Ph.D., Prof.[1,2,3,4,+]**, Yu-Chieh Chuang**, M.D.[5,6,+]**,**
**Edwin Chan**, Ph.D., Prof.[7,8,9]**, Jin-Hua Chen,** Ph.D., Prof. [1,10,11,12]**,**
**Wen-Hsuan Hou**, M.D., Ph.D., Prof.[1,13,14+]**, Enoch Kang,** M.A., consultant [1,2,15,*] **,**
**Cochrane Taiwan**[#]

1. Cochrane Taiwan, Taipei Medical University, Taipei, Taiwan
2. Evidence-Based Medicine Center, Wan Fang Hospital, Taipei Medical University, Taipei, Taiwan
3. Department of Public Health, School of Medicine, College of Medicine, Taipei Medical University, Taipei, Taiwan
4. Division of Plastic Surgery, Department of Surgery, Wan Fang Hospital, Taipei Medical University, Taipei, Taiwan
5. Department of Psychiatry, Taipei City Psychiatric Center, Taipei City Hospital, Songde branch, Taipe, Taiwan
6. School of Medicine, College of Medicine, Taipei Medical University, Taipei, Taiwan
7. Cochrane Singapore, Singapore, Singapore
8. Singapore Clinical Research Institute, Singapore, Singapore
9. Duke-NUS Medical School, Singapore, Singapore
10. Graduate Institute of Data Science, College of Management, Taipei Medical University, Taipei 110, Taiwan.
11. Research Center of Biostatistics Center, College of Management, Taipei Medical University, Taipei 110, Taiwan.
12. Biostatistics Center, Wan Fang Hospital, Taipei Medical University, Taipei 116, Taiwan.
13. College of Medicine, National Cheng Kung University, Tainan, Taiwan
14. Department of Geriatrics and Gerontology, National Cheng Kung University Hospital, College of Medicine, National Cheng Kung University, Tainan, Taiwan
15. Institute of Health Policy & Management, College of Public Health, National Taiwan University, Taipei, Taiwan

# Content

**Figure S1** Reproduced results of network meta-analysis.

**Figure S2** Series of line charts of probability of every surgical procedure on each possible rank for fusion rate.

**Figure S3** Multi-line plot of probability of every surgical procedure on each possible rank for fusion rate.

**Figure S4** Stacked bar chart of probability of every surgical procedure on each possible rank for fusion rate.

**Figure S5** Series of line charts of cumulative probability of every surgical procedure on possible rank for fusion rate.

**Figure S6** Multi-line plot of cumulative probability of every surgical procedure on possible rank for fusion rate.

**Figure S7** Simple bar charts of global metrics of treatment rankings on fusion rate.

**Figure S8** Simple line charts of global metrics of treatment rankings on fusion rate.

**Figure S9** Heat plots of global metrics of treatment rankings on fusion rate.

**Figure S10** Series of line charts of probability of every surgical procedure on each possible rank for Oswestry Disability Index.

**Figure S11** Multi-line plot of probability of every surgical procedure on each possible rank for Oswestry Disability Index.

**Figure S12** Stacked bar chart of probability of every surgical procedure on each possible rank for Oswestry Disability Index.

**Figure S13** Series of line charts of cumulative probability of every surgical procedure on possible rank for Oswestry Disability Index.

**Figure S14** Multi-line plot of cumulative probability of every surgical procedure on possible rank for Oswestry Disability Index.

**Figure S15** Simple bar charts of global metrics of treatment rankings on Oswestry Disability Index.

**Figure S16** Simple line charts of global metrics of treatment rankings on Oswestry Disability Index.

**Figure S17** Heat plots of global metrics of treatment rankings on Oswestry Disability Index.

**Figure S18** Series of line charts of probability of every surgical procedure on each possible rank for adverse event.

**Figure S19** Multi-line plot of probability of every surgical procedure on each possible rank for adverse event.

**Figure S20** Stacked bar chart of probability of every surgical procedure on each possible rank for adverse event.

**Figure S21** Series of line charts of cumulative probability of every surgical procedure on possible rank for adverse event.

**Figure S22** Multi-line plot of cumulative probability of every surgical procedure on possible rank for adverse event.

**Figure S23** Simple bar charts of global metrics of treatment rankings on adverse event.

**Figure S24** Simple line charts of global metrics of treatment rankings on adverse event.

**Figure S25** Heat plots of global metrics of treatment rankings on adverse event.

**Figure S26** Series of line charts of probability of every surgical procedure on each possible rank for operative time.

**Figure S27** Multi-line plot of probability of every surgical procedure on each possible rank for operative time.

**Figure S28** Stacked bar chart of probability of every surgical procedure on each possible rank for operative time.

**Figure S29** Series of line charts of cumulative probability of every surgical procedure on possible rank for operative time.

**Figure S30** Multi-line plot of cumulative probability of every surgical procedure on possible rank for operative time.

**Figure S31** Simple bar charts of global metrics of treatment rankings on operative time.

**Figure S32** Simple line charts of global metrics of treatment rankings on operative time.

**Figure S33** Heat plots of global metrics of treatment rankings on operative time.

**Figure S34** Spie plot of network meta-analysis.

**Figure S35** Rank-heat plot of network meta-analysis on four outcomes using SUCRA.

**Figure S36** Scatter plot of global metrics of treatment rankings.

**Figure S37** Colorblind-friendly beading plot of global metrics of treatment rankings by SCURA

**Table S1** Further information for beading plot

**File S1** Steps and R code for generating beading plot

**Figure S1** Reproduced results of network meta-analysis on (A) fusion rate, (B) Oswestry Disability Index, (C) adverse event, and (D) operative time. CI, confidence interval; MD, mean difference; MTLIF, minimally invasive transforaminal interbody fusion; PLF, posterolateral fusion; PLIF, posterior lumbar interbody fusion; RR, risk ratio; TLIF, transforaminal interbody fusion; XLIF, extreme lateral interbody fusion.

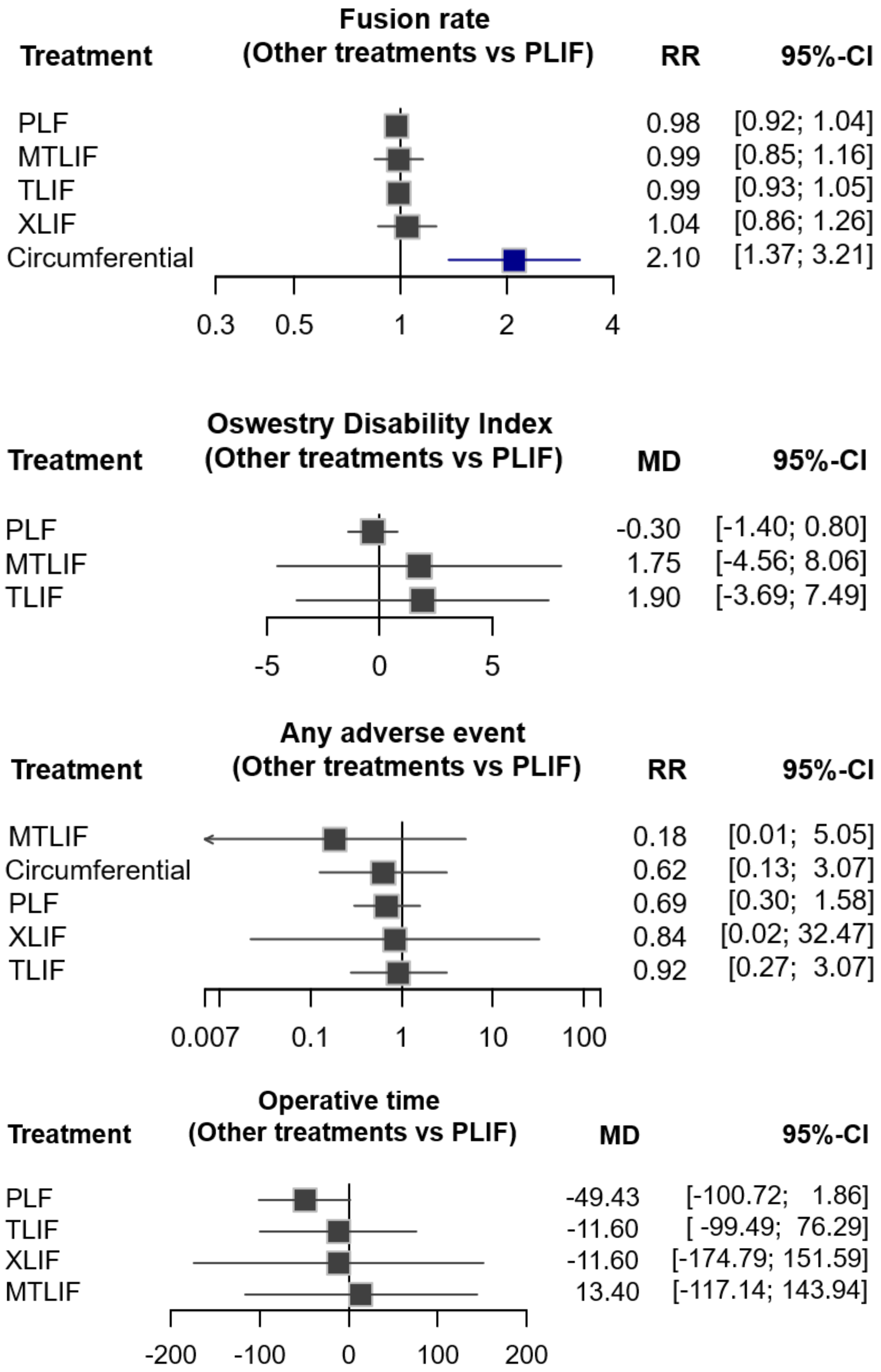


(A)

(B)

(C)

(D)

**Figure S2** Series of line charts of probability of every surgical procedure on each possible rank for fusion rate. MTLIF, minimally invasive transforaminal interbody fusion; PLF, posterolateral fusion; PLIF, posterior lumbar interbody fusion; TLIF, transforaminal interbody fusion; XLIF, extreme lateral interbody fusion.

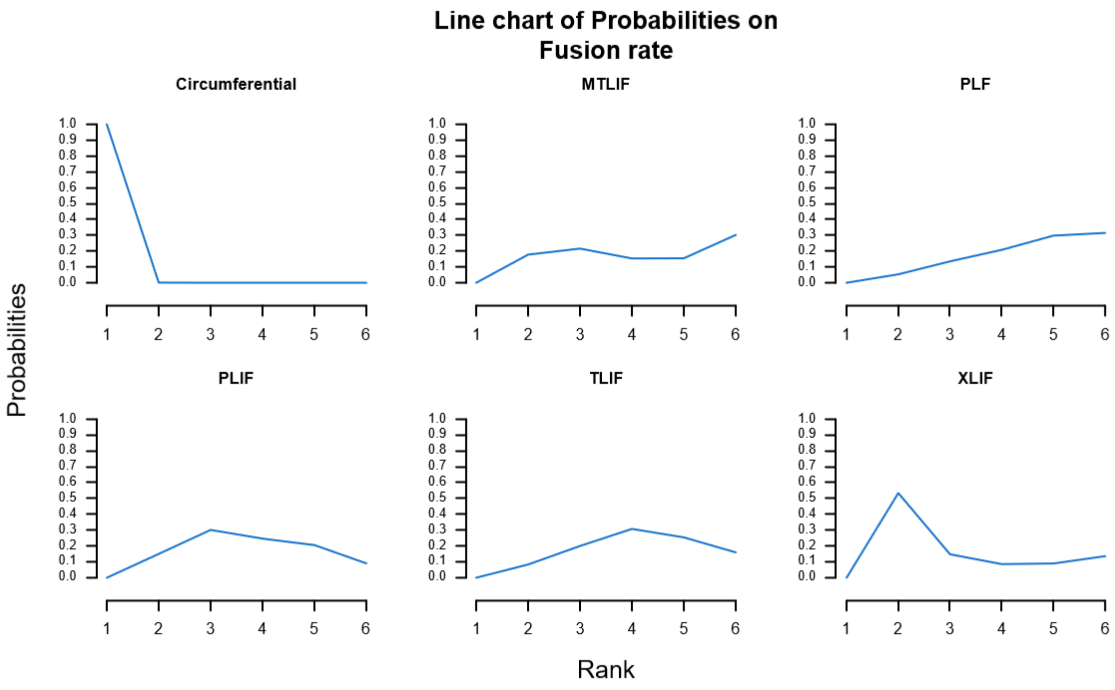


**Figure S3** Multi-line plot of probability of every surgical procedure on each possible rank for fusion rate. MTLIF, minimally invasive transforaminal interbody fusion; PLF, posterolateral fusion; PLIF, posterior lumbar interbody fusion; TLIF, transforaminal interbody fusion; XLIF, extreme lateral interbody fusion.

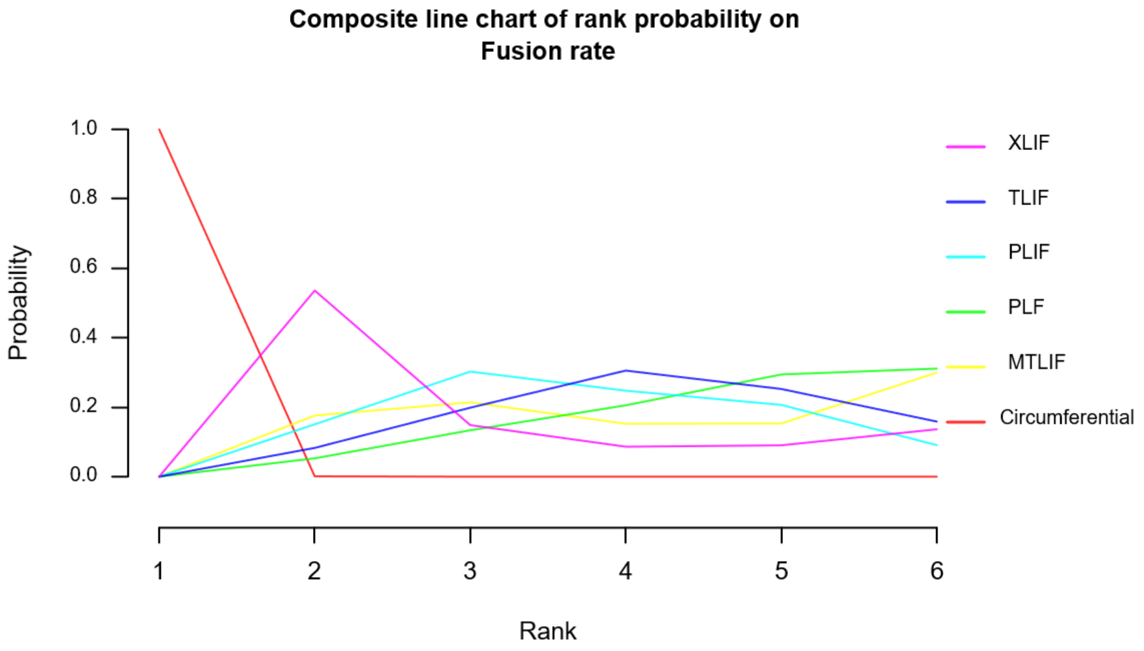

**Figure S4** Stacked bar chart of probability of every surgical procedure on each possible rank for fusion rate. MTLIF, minimally invasive transforaminal interbody fusion; PLF, posterolateral fusion; PLIF, posterior lumbar interbody fusion; TLIF, transforaminal interbody fusion; XLIF, extreme lateral interbody fusion.

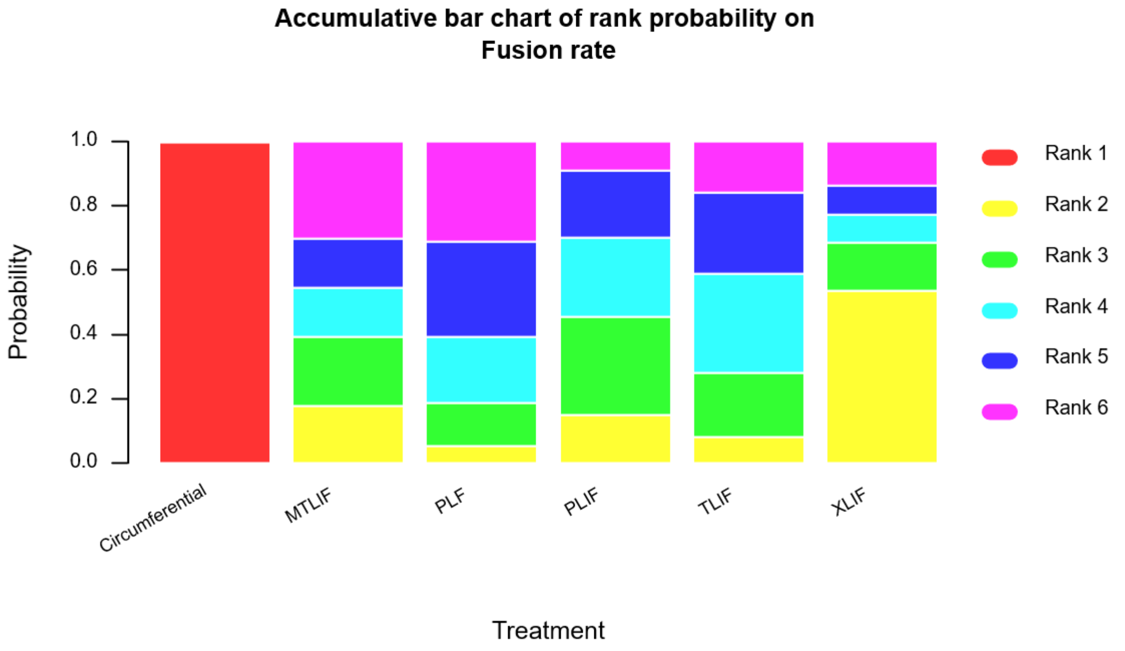

**Figure S5** Series of line charts of cumulative probability of every surgical procedure on possible rank for fusion rate. MTLIF, minimally invasive transforaminal interbody fusion; PLF, posterolateral fusion; PLIF, posterior lumbar interbody fusion; TLIF, transforaminal interbody fusion; XLIF, extreme lateral interbody fusion.

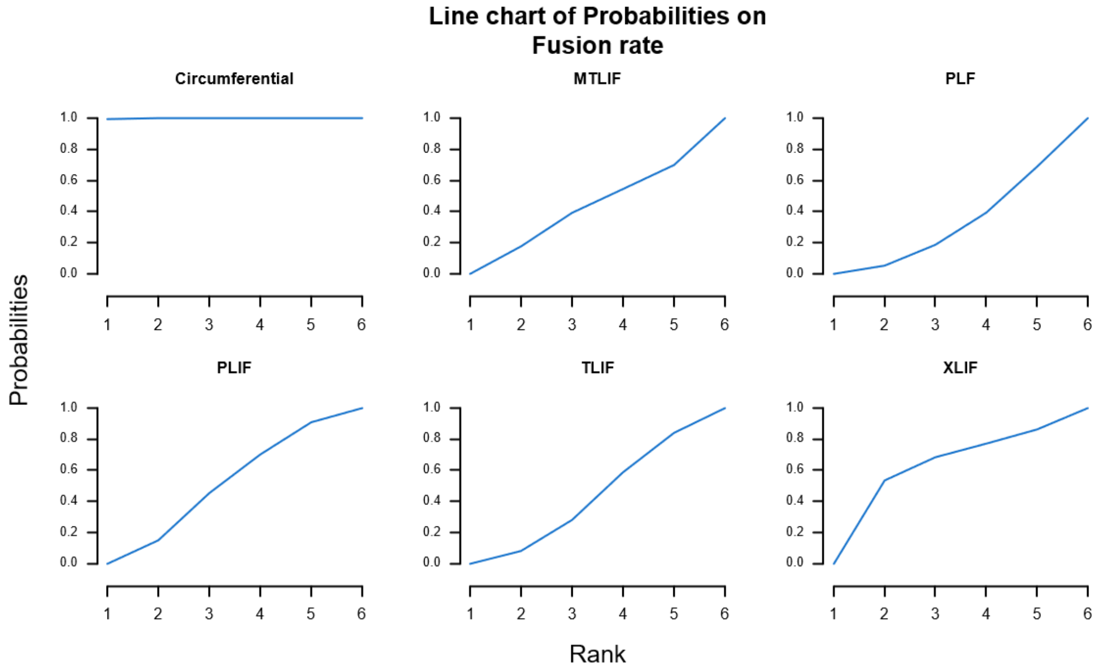


**Figure S6** Multi-line plot of cumulative probability of every surgical procedure on possible rank for fusion rate. MTLIF, minimally invasive transforaminal interbody fusion; PLF, posterolateral fusion; PLIF, posterior lumbar interbody fusion; TLIF, transforaminal interbody fusion; XLIF, extreme lateral interbody fusion.

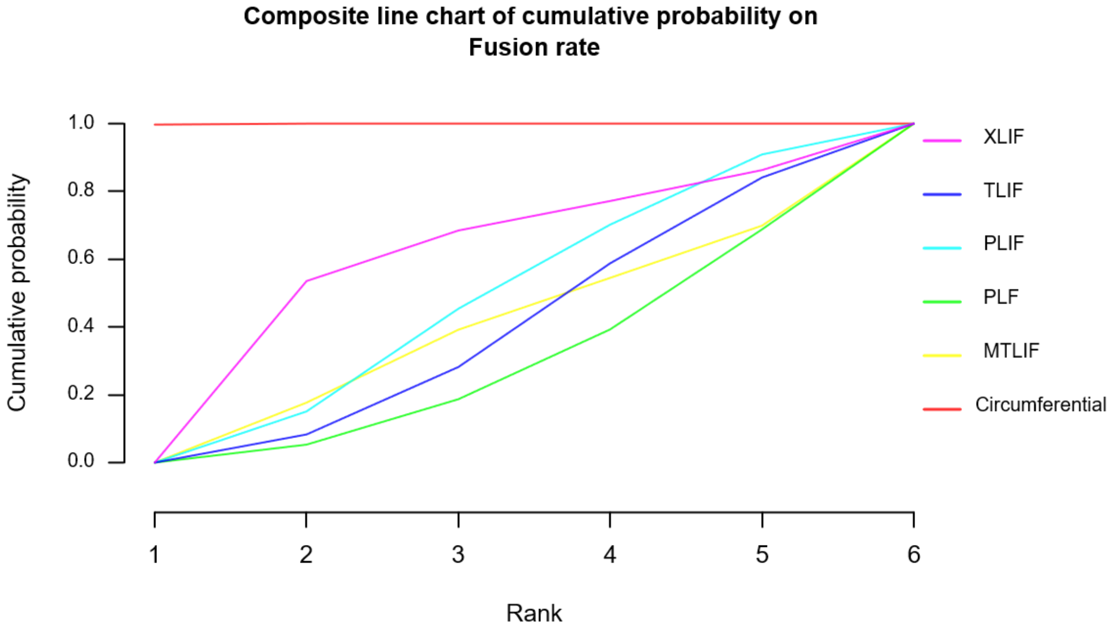

**Figure S7** Simple bar charts of global metrics of treatment rankings on fusion rate using (A) P-score and (B) SUCRA. MTLIF, minimally invasive transforaminal interbody fusion; PLF, posterolateral fusion; PLIF, posterior lumbar interbody fusion; TLIF, transforaminal interbody fusion; XLIF, extreme lateral interbody fusion.

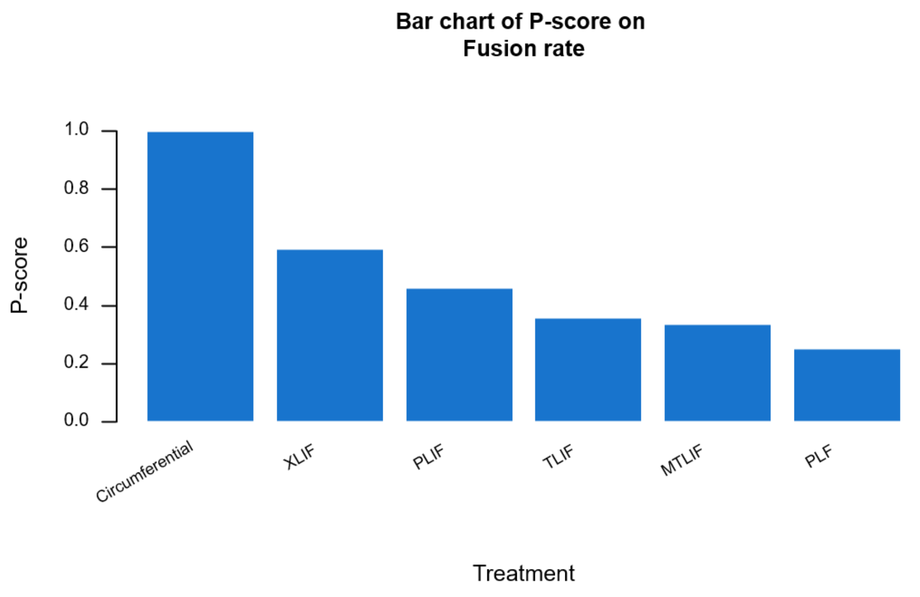


(A)

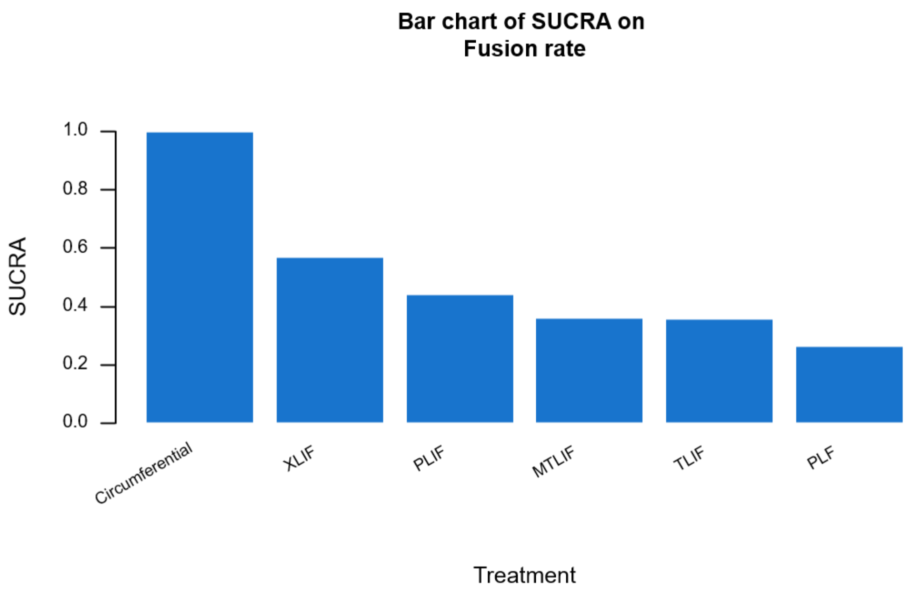


(B)

**Figure S8** Simple line charts of global metrics of treatment rankings on fusion rate using (A) P-score and (B) SUCRA. MTLIF, minimally invasive transforaminal interbody fusion; PLF, posterolateral fusion; PLIF, posterior lumbar interbody fusion; TLIF, transforaminal interbody fusion; XLIF, extreme lateral interbody fusion.

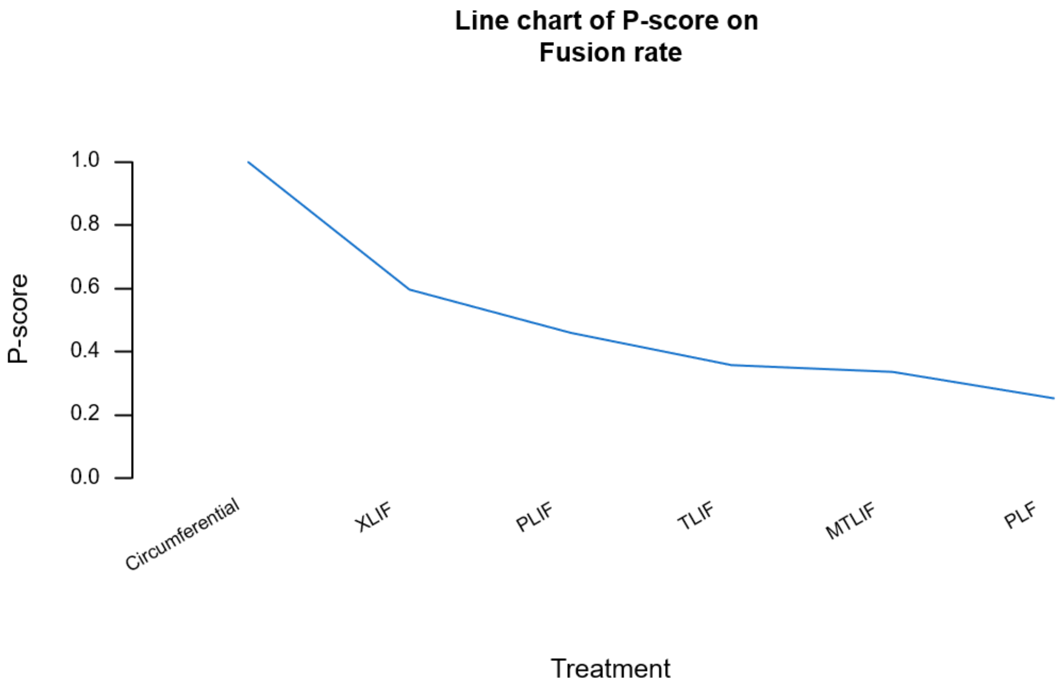


(A)

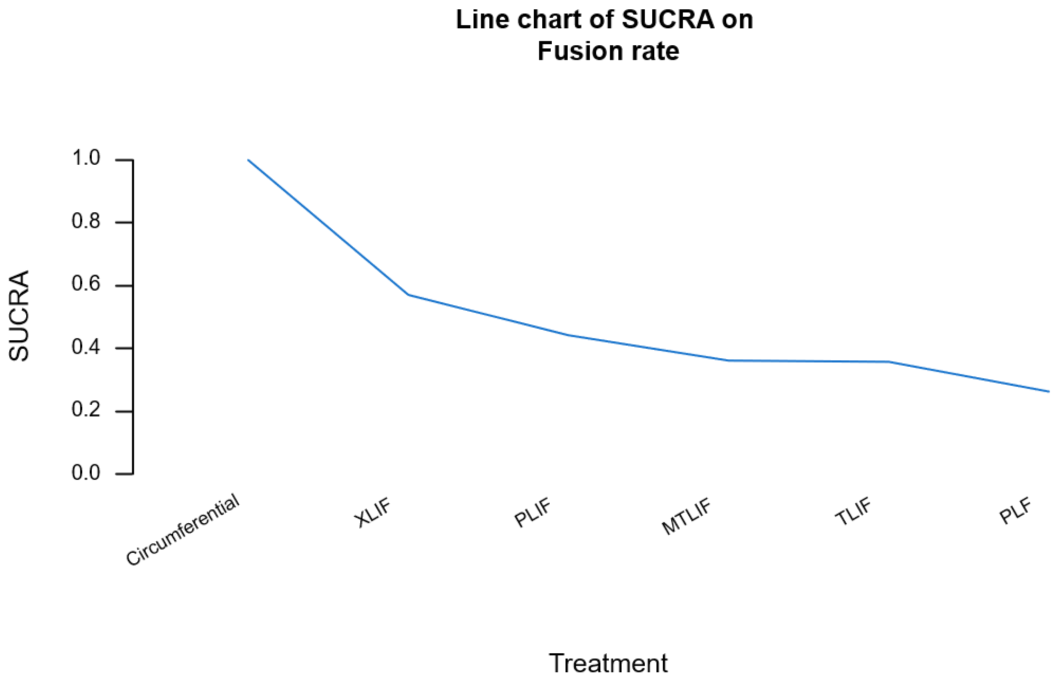


(B)

**Figure S9** Heat plots of global metrics of treatment rankings on fusion rate using (A) P-score and (B) SUCRA. MTLIF, minimally invasive transforaminal interbody fusion; PLF, posterolateral fusion; PLIF, posterior lumbar interbody fusion; TLIF, transforaminal interbody fusion; XLIF, extreme lateral interbody fusion.

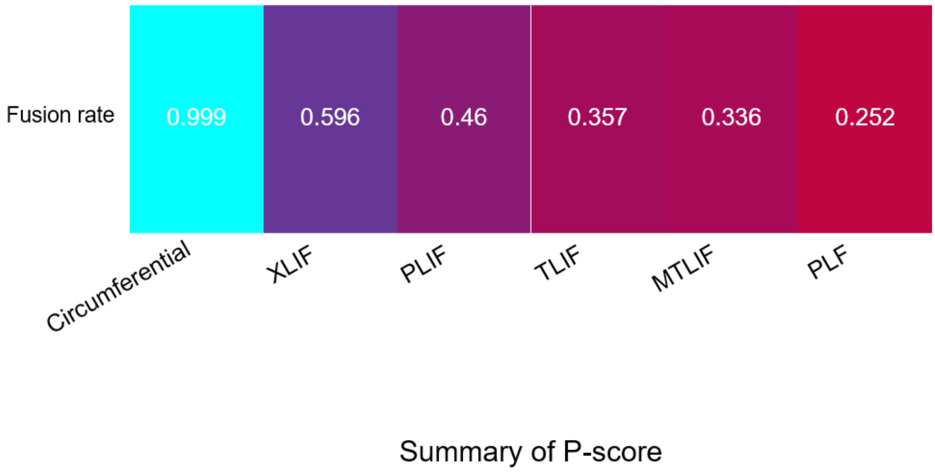


(A)

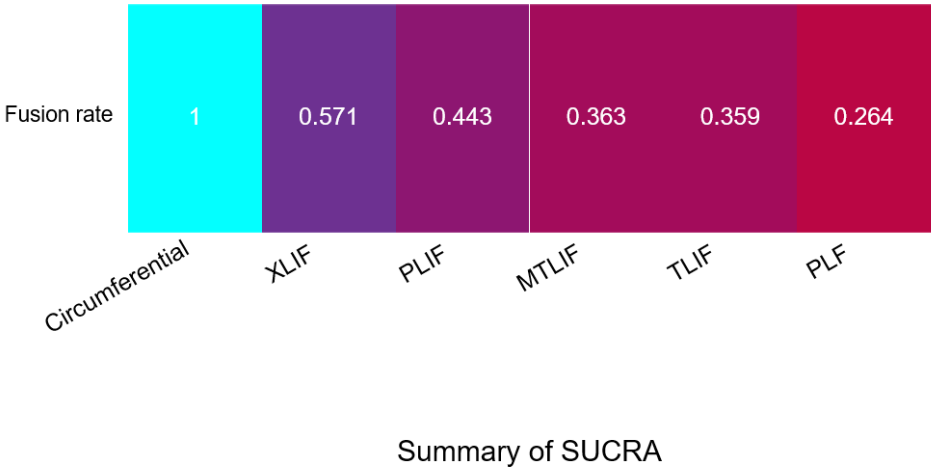


(B)

**Figure S10** Series of line charts of probability of every surgical procedure on each possible rank for Oswestry Disability Index. MTLIF, minimally invasive transforaminal interbody fusion; PLF, posterolateral fusion; PLIF, posterior lumbar interbody fusion; TLIF, transforaminal interbody fusion.

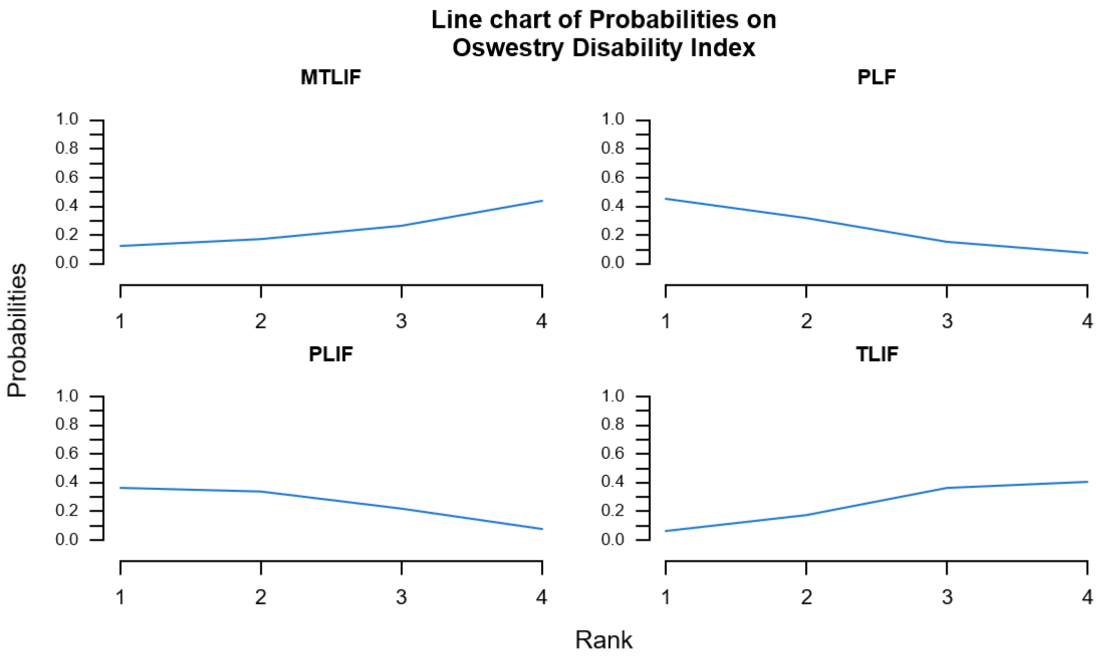


**Figure S11** Multi-line plot of probability of every surgical procedure on each possible rank for Oswestry Disability Index. MTLIF, minimally invasive transforaminal interbody fusion; PLF, posterolateral fusion; PLIF, posterior lumbar interbody fusion; TLIF, transforaminal interbody fusion.

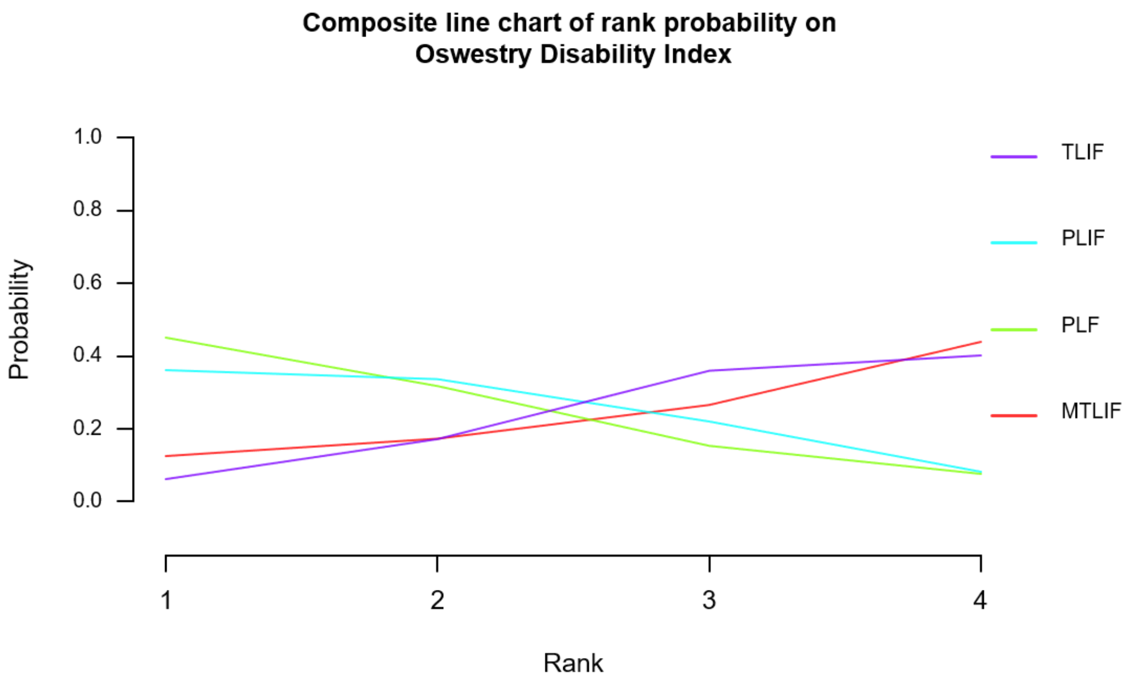

**Figure S12** Stacked bar chart of probability of every surgical procedure on each possible rank for Oswestry Disability Index. MTLIF, minimally invasive transforaminal interbody fusion; PLF, posterolateral fusion; PLIF, posterior lumbar interbody fusion; TLIF, transforaminal interbody fusion.

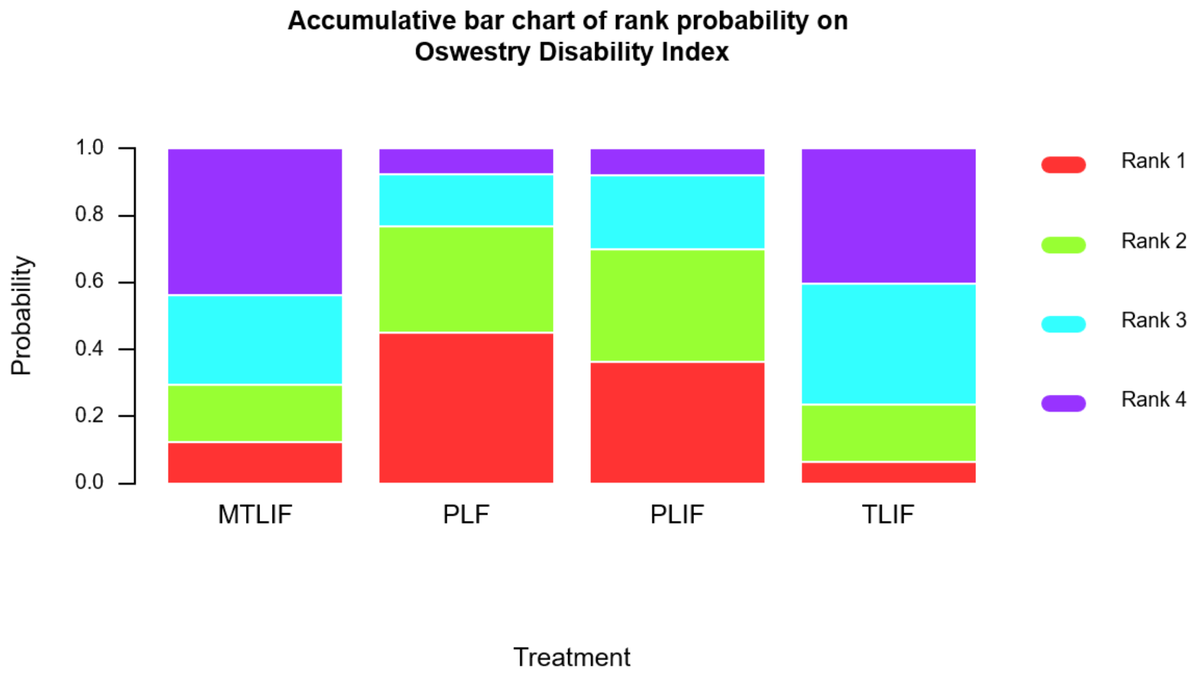

**Figure S13** Series of line charts of cumulative probability of every surgical procedure on possible rank for Oswestry Disability Index. MTLIF, minimally invasive transforaminal interbody fusion; PLF, posterolateral fusion; PLIF, posterior lumbar interbody fusion; TLIF, transforaminal interbody fusion.

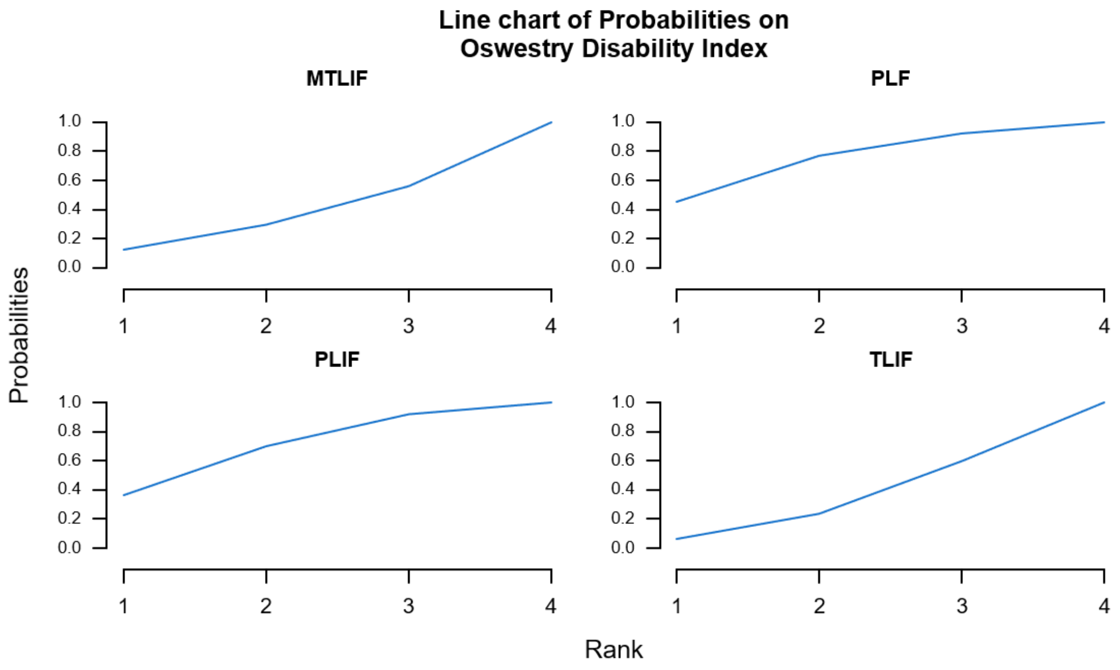


**Figure S14** Multi-line plot of cumulative probability of every surgical procedure on possible rank for Oswestry Disability Index. MTLIF, minimally invasive transforaminal interbody fusion; PLF, posterolateral fusion; PLIF, posterior lumbar interbody fusion; TLIF, transforaminal interbody fusion.

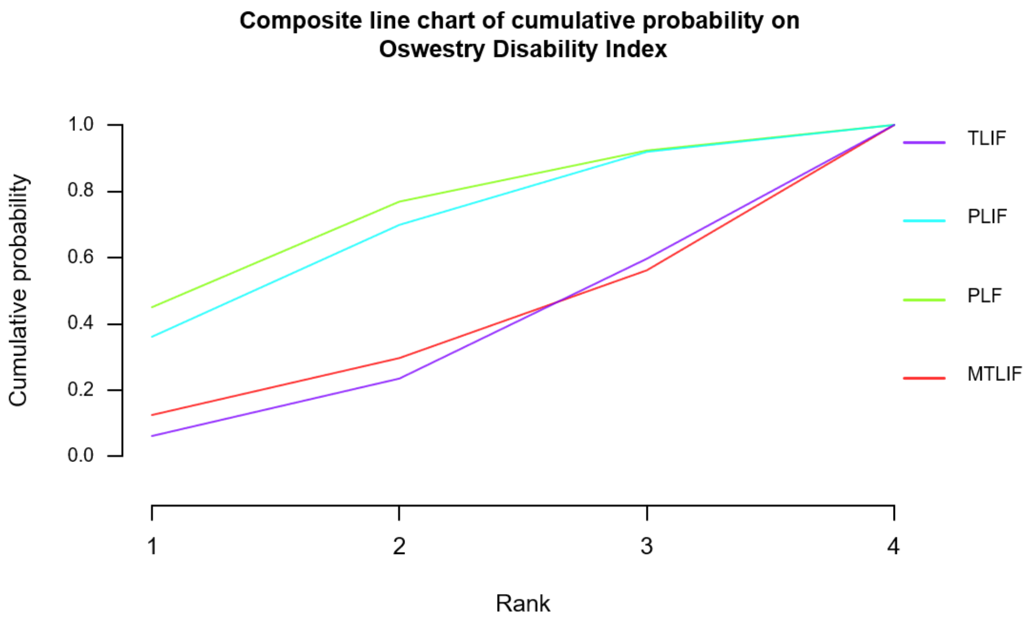

**Figure S15** Simple bar charts of global metrics of treatment rankings on Oswestry Disability Index using (A) P-score and (B) SUCRA. MTLIF, minimally invasive transforaminal interbody fusion; PLF, posterolateral fusion; PLIF, posterior lumbar interbody fusion; TLIF, transforaminal interbody fusion.

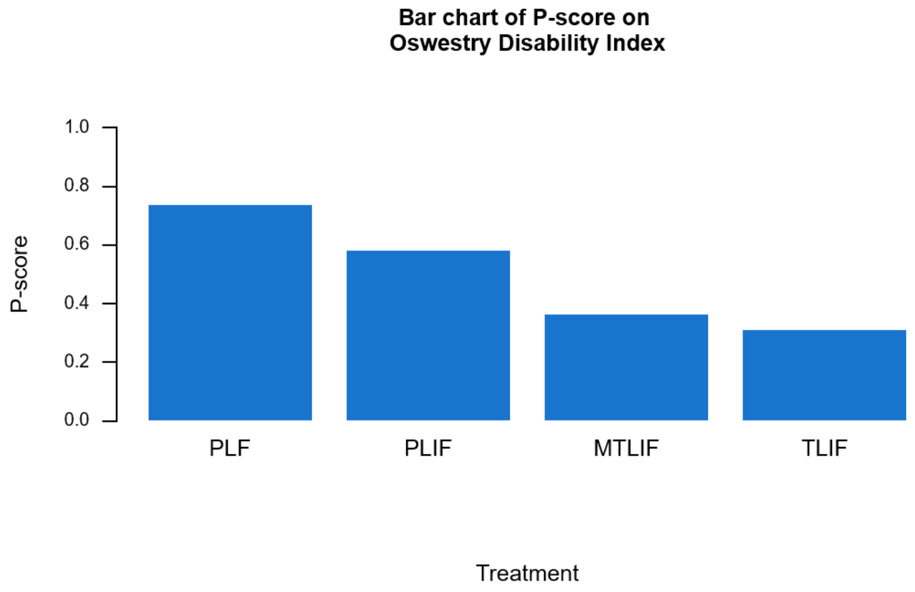


(A)

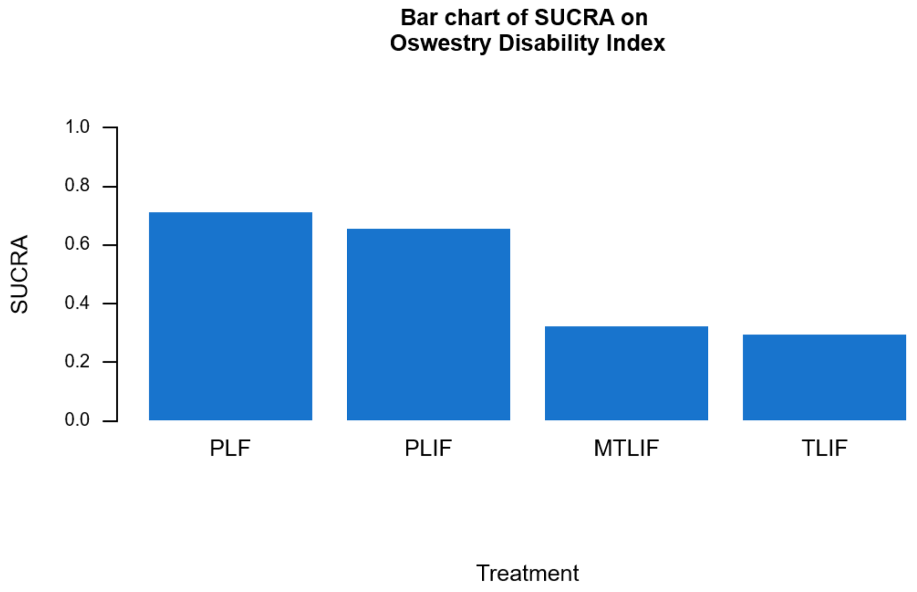


(B)

**Figure S16** Simple line charts of global metrics of treatment rankings on Oswestry Disability Index using (A) P-score and (B) SUCRA. MTLIF, minimally invasive transforaminal interbody fusion; PLF, posterolateral fusion; PLIF, posterior lumbar interbody fusion; TLIF, transforaminal interbody fusion.

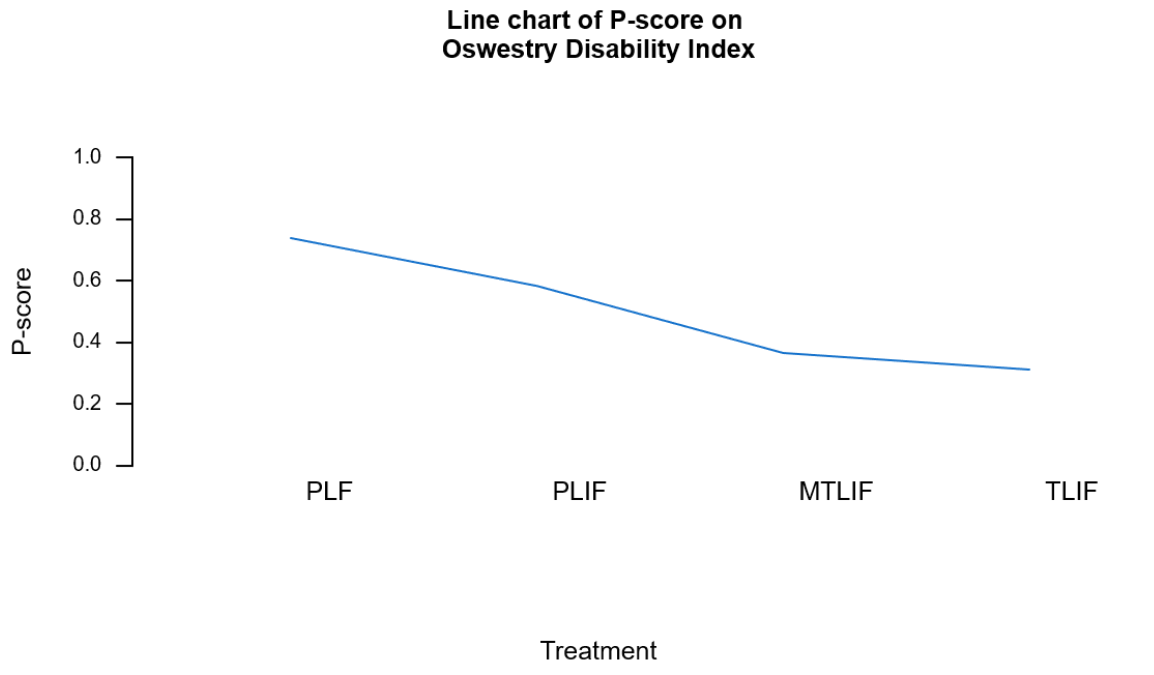


(A)

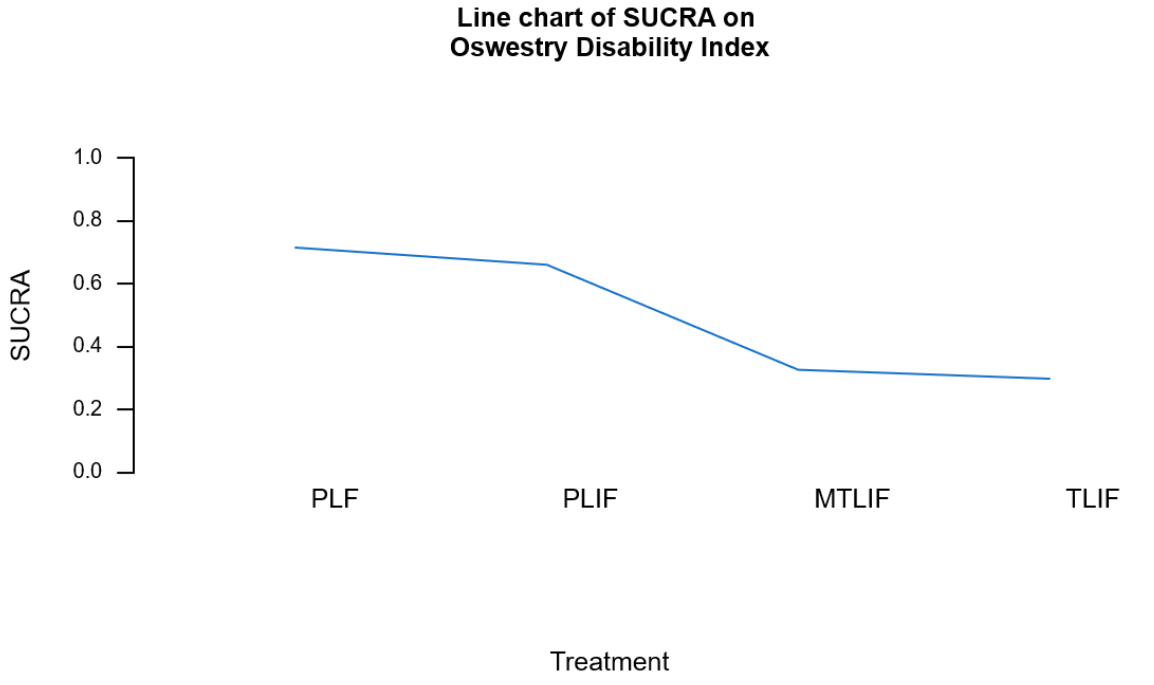


(B)

**Figure S17** Heat plots of global metrics of treatment rankings on Oswestry Disability Index using (A) P-score and (B) SUCRA. MTLIF, minimally invasive transforaminal interbody fusion; PLF, posterolateral fusion; PLIF, posterior lumbar interbody fusion; TLIF, transforaminal interbody fusion.

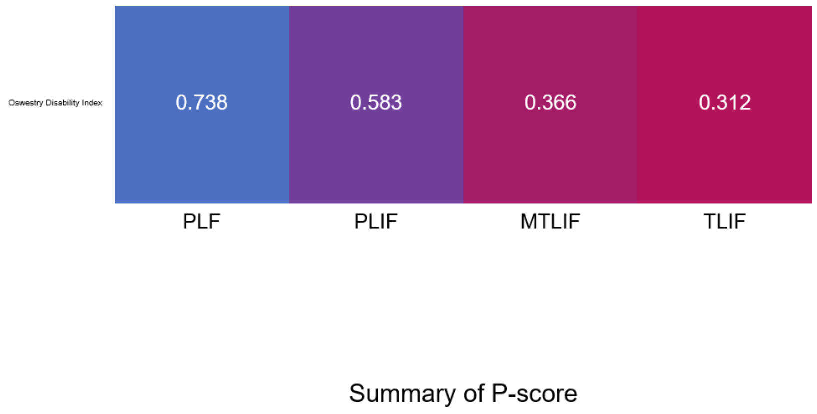


(A)

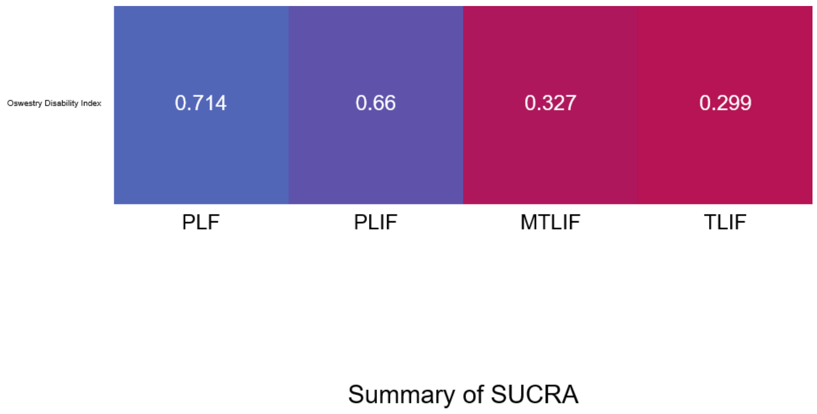


(B)

**Figure S18** Series of line charts of probability of every surgical procedure on each possible rank for adverse event. MTLIF, minimally invasive transforaminal interbody fusion; PLF, posterolateral fusion; PLIF, posterior lumbar interbody fusion; TLIF, transforaminal interbody fusion; XLIF, extreme lateral interbody fusion.

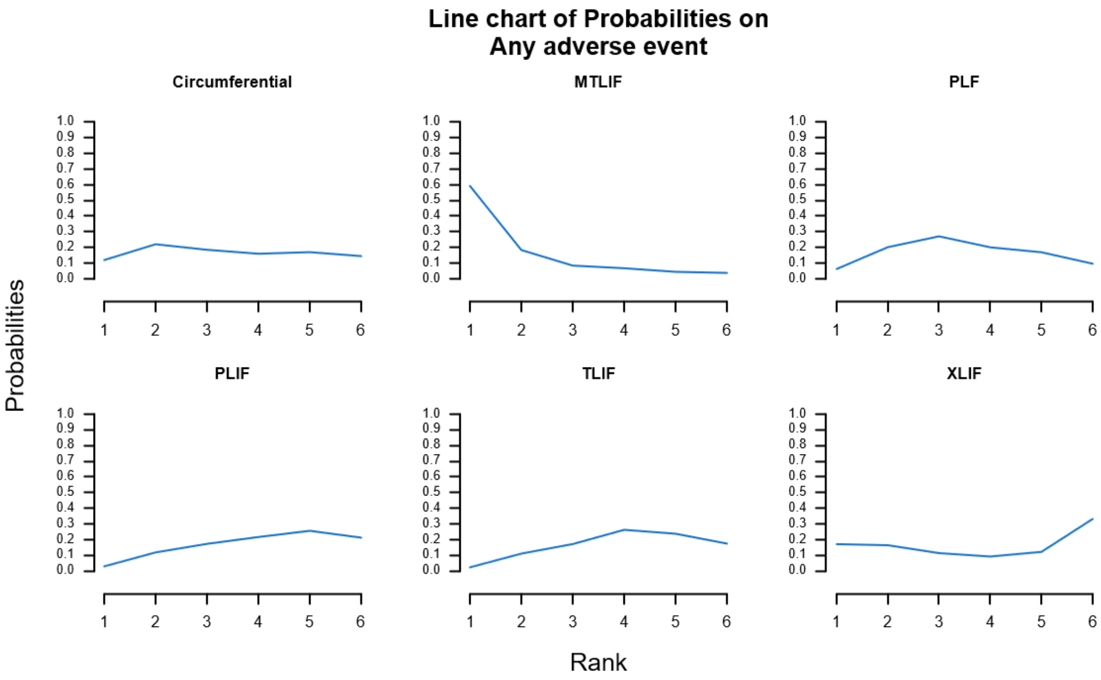


**Figure S19** Multi-line plot of probability of every surgical procedure on each possible rank for adverse event. MTLIF, minimally invasive transforaminal interbody fusion; PLF, posterolateral fusion; PLIF, posterior lumbar interbody fusion; TLIF, transforaminal interbody fusion; XLIF, extreme lateral interbody fusion.

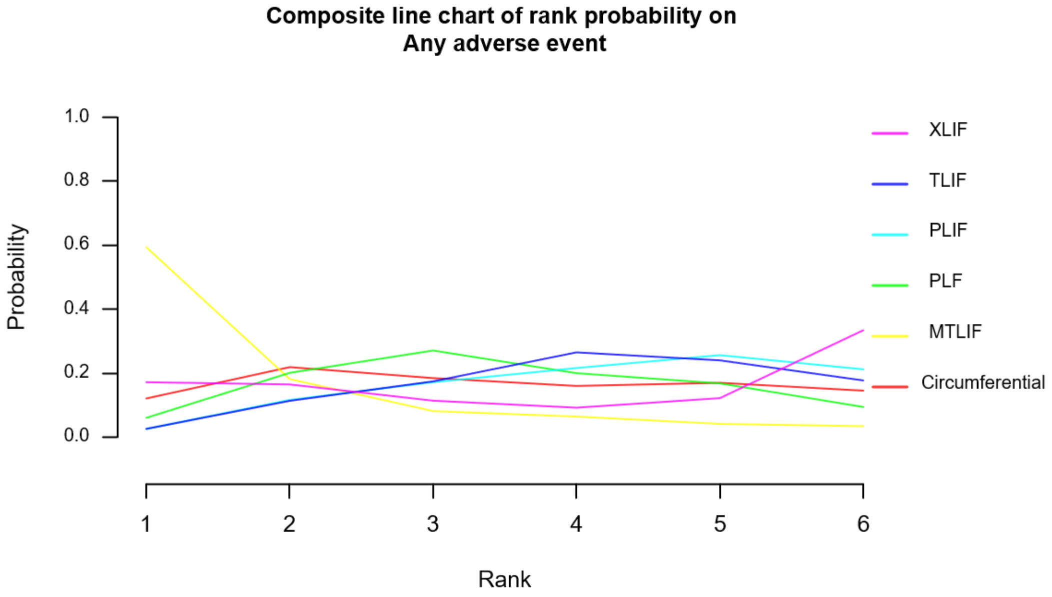

**Figure S20** Stacked bar chart of probability of every surgical procedure on each possible rank for adverse event. MTLIF, minimally invasive transforaminal interbody fusion; PLF, posterolateral fusion; PLIF, posterior lumbar interbody fusion; TLIF, transforaminal interbody fusion; XLIF, extreme lateral interbody fusion.

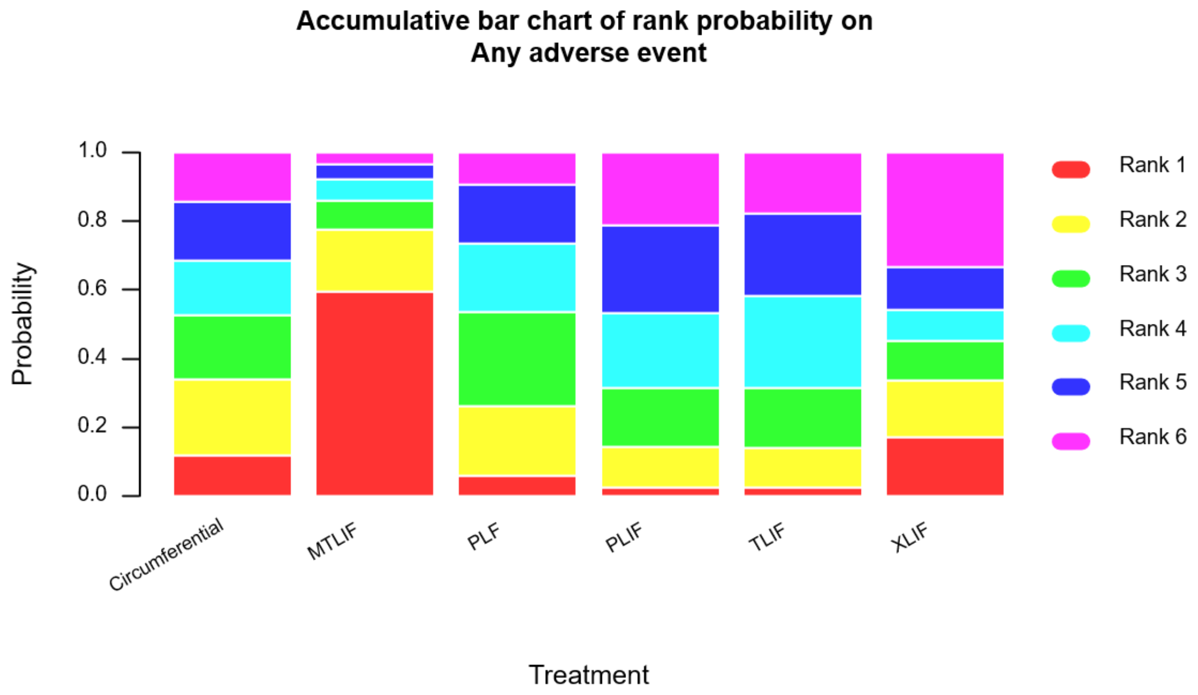

**Figure S21** Series of line charts of cumulative probability of every surgical procedure on possible rank for adverse event. MTLIF, minimally invasive transforaminal interbody fusion; PLF, posterolateral fusion; PLIF, posterior lumbar interbody fusion; TLIF, transforaminal interbody fusion; XLIF, extreme lateral interbody fusion.

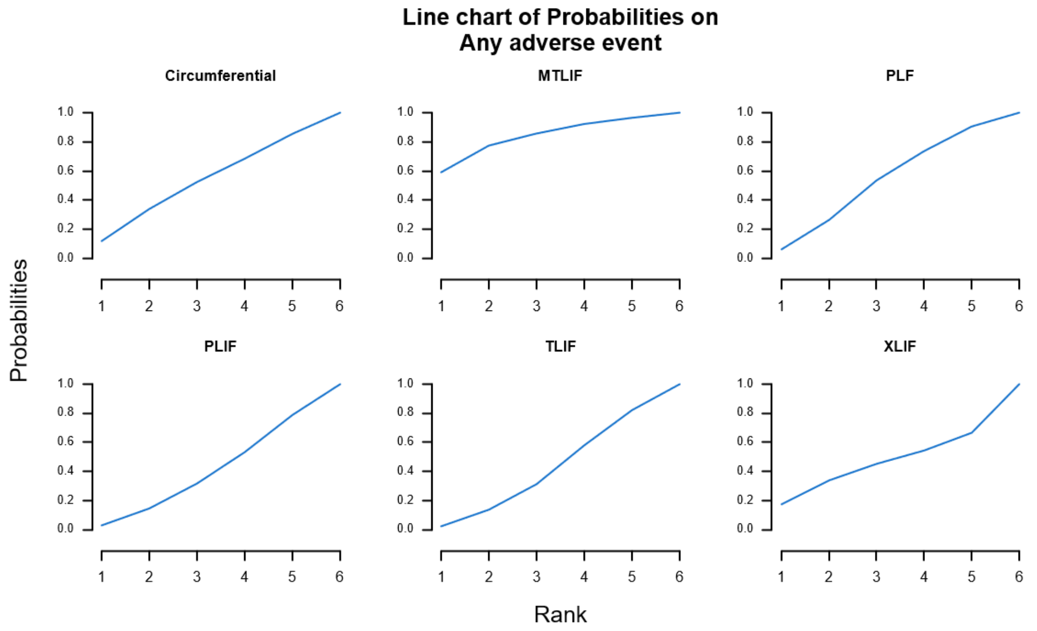


**Figure S22** Multi-line plot of cumulative probability of every surgical procedure on possible rank for adverse event. MTLIF, minimally invasive transforaminal interbody fusion; PLF, posterolateral fusion; PLIF, posterior lumbar interbody fusion; TLIF, transforaminal interbody fusion; XLIF, extreme lateral interbody fusion.

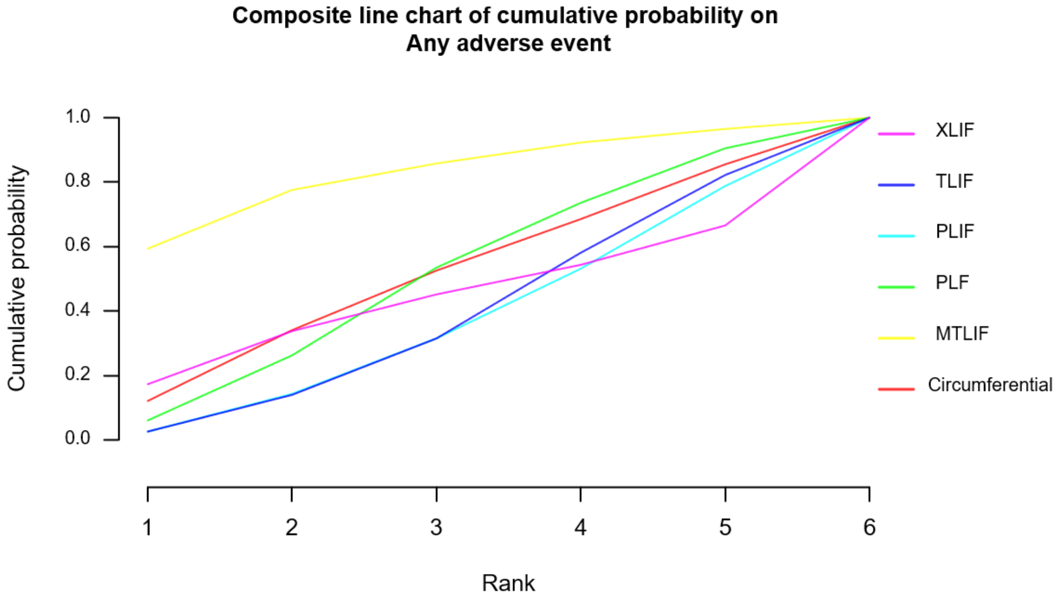

**Figure S23** Simple bar charts of global metrics of treatment rankings on adverse event using (A) P-score and (B) SUCRA. MTLIF, minimally invasive transforaminal interbody fusion; PLF, posterolateral fusion; PLIF, posterior lumbar interbody fusion; TLIF, transforaminal interbody fusion; XLIF, extreme lateral interbody fusion.

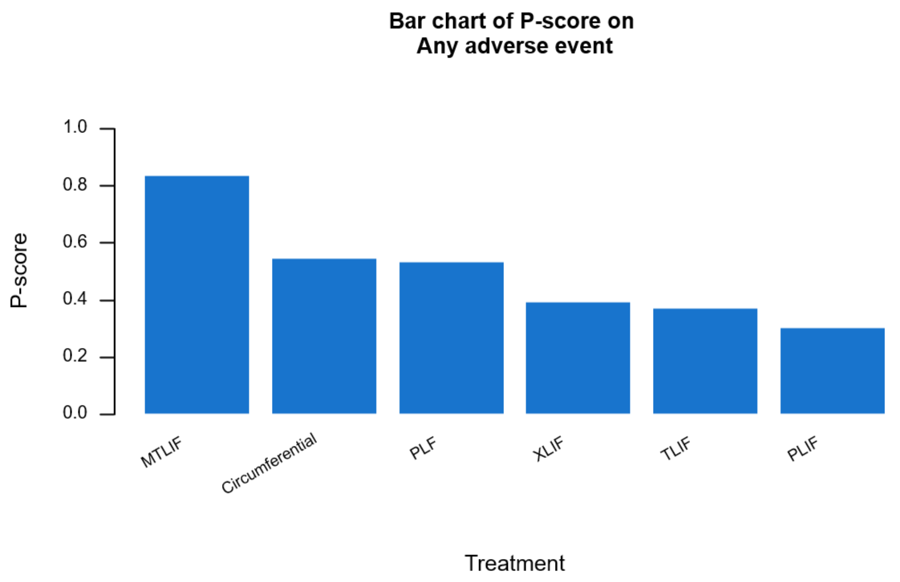


(A)

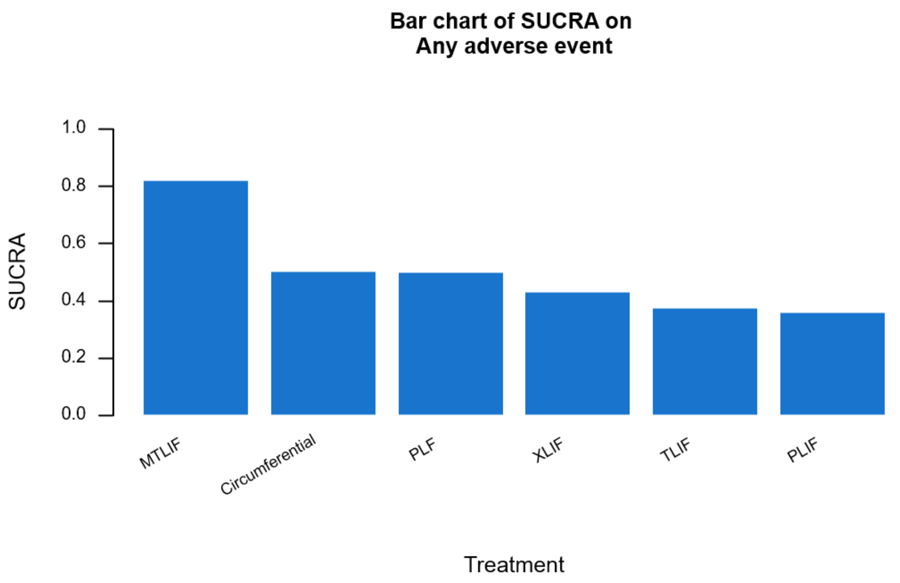


(B)

**Figure S24** Simple line charts of global metrics of treatment rankings on adverse event using (A) P-score and (B) SUCRA. MTLIF, minimally invasive transforaminal interbody fusion; PLF, posterolateral fusion; PLIF, posterior lumbar interbody fusion; TLIF, transforaminal interbody fusion; XLIF, extreme lateral interbody fusion.

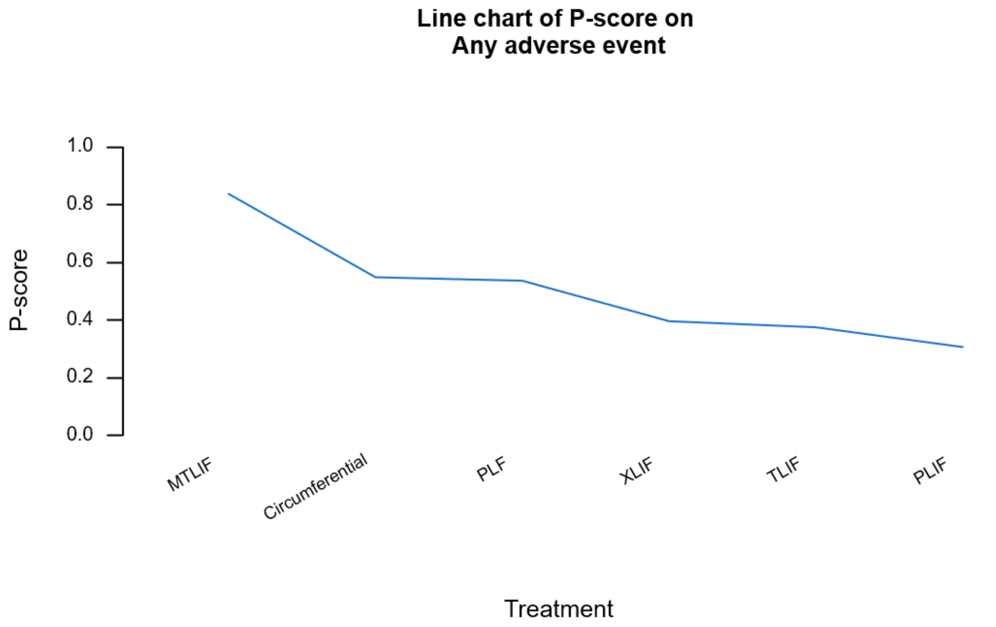


(A)

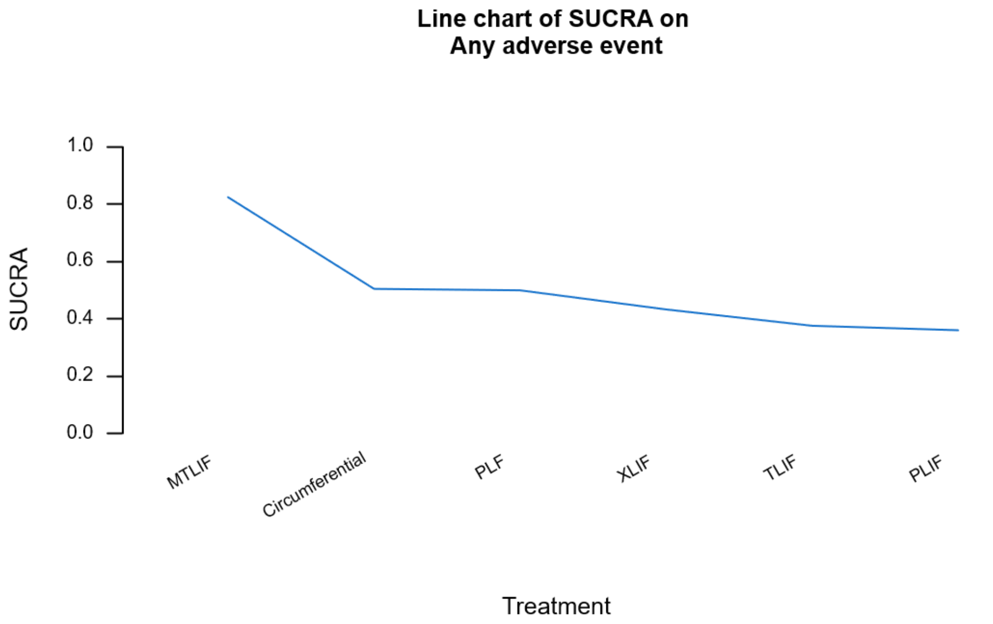


(B)

**Figure S25** Heat plots of global metrics of treatment rankings on adverse event using (A) P-score and (B) SUCRA. MTLIF, minimally invasive transforaminal interbody fusion; PLF, posterolateral fusion; PLIF, posterior lumbar interbody fusion; TLIF, transforaminal interbody fusion; XLIF, extreme lateral interbody fusion.

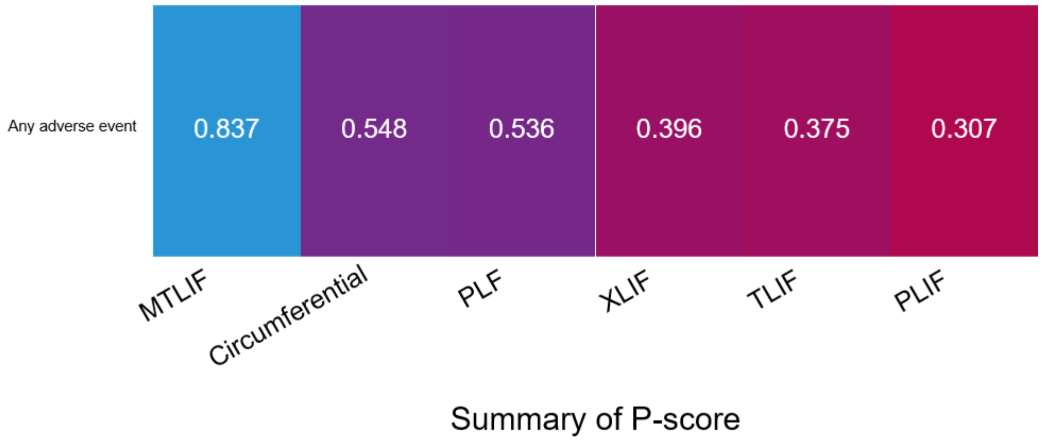


(A)

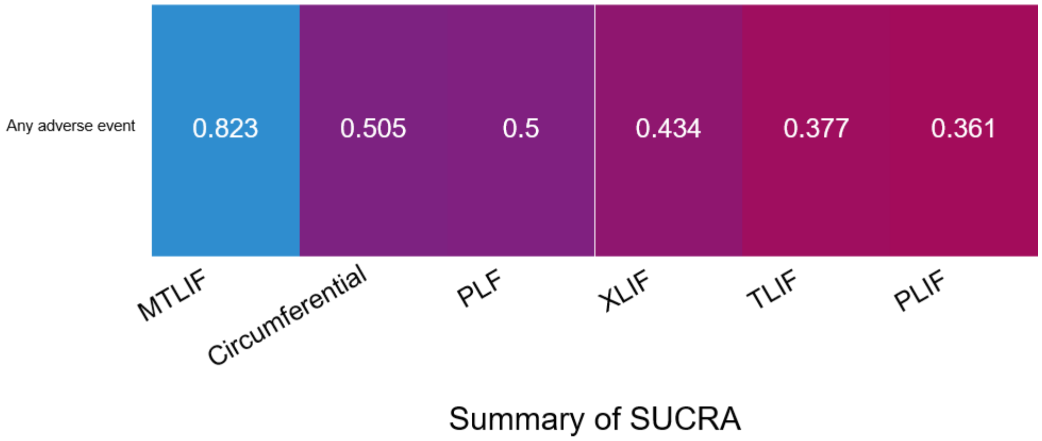


(B)

**Figure S26** Series of line charts of probability of every surgical procedure on each possible rank for operative time. MTLIF, minimally invasive transforaminal interbody fusion; PLF, posterolateral fusion; PLIF, posterior lumbar interbody fusion; TLIF, transforaminal interbody fusion; XLIF, extreme lateral interbody fusion.

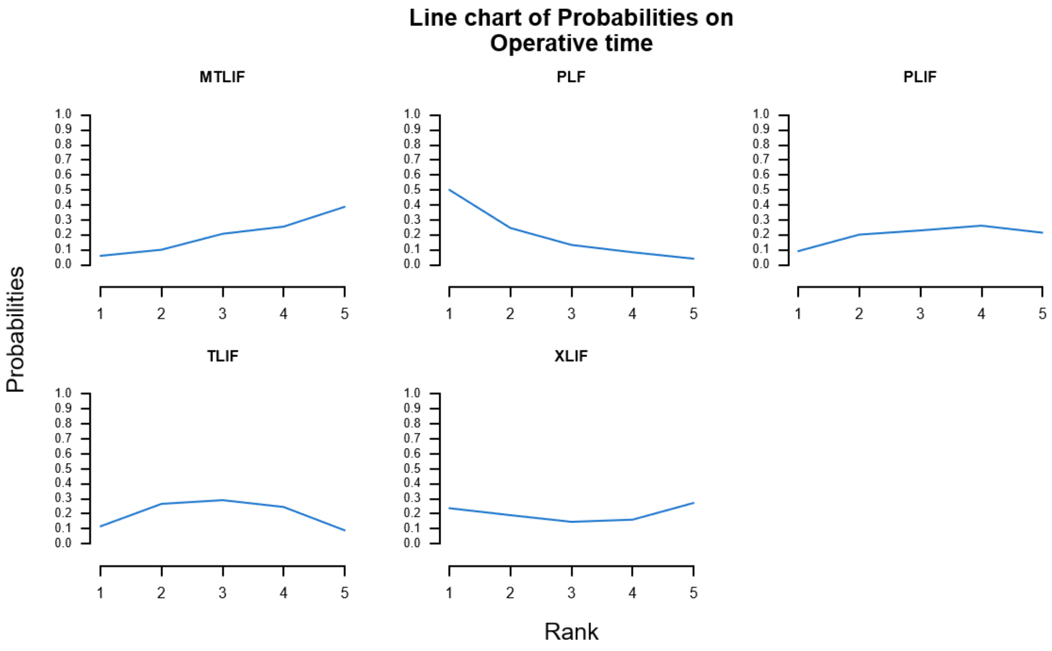


**Figure S27** Multi-line plot of probability of every surgical procedure on each possible rank for operative time. MTLIF, minimally invasive transforaminal interbody fusion; PLF, posterolateral fusion; PLIF, posterior lumbar interbody fusion; TLIF, transforaminal interbody fusion; XLIF, extreme lateral interbody fusion.

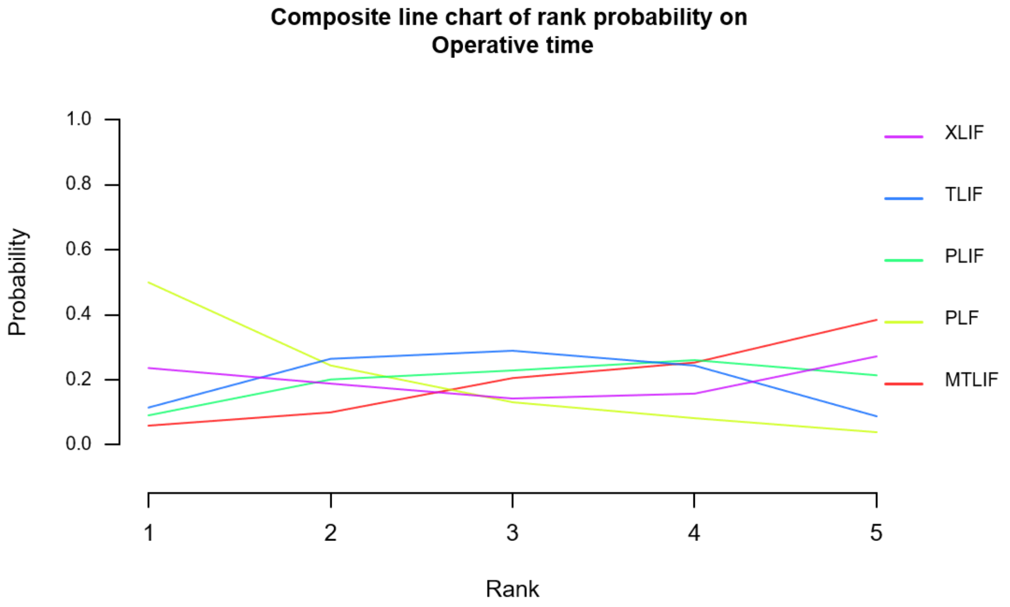

**Figure S28** Stacked bar chart of probability of every surgical procedure on each possible rank for operative time. MTLIF, minimally invasive transforaminal interbody fusion; PLF, posterolateral fusion; PLIF, posterior lumbar interbody fusion; TLIF, transforaminal interbody fusion; XLIF, extreme lateral interbody fusion.

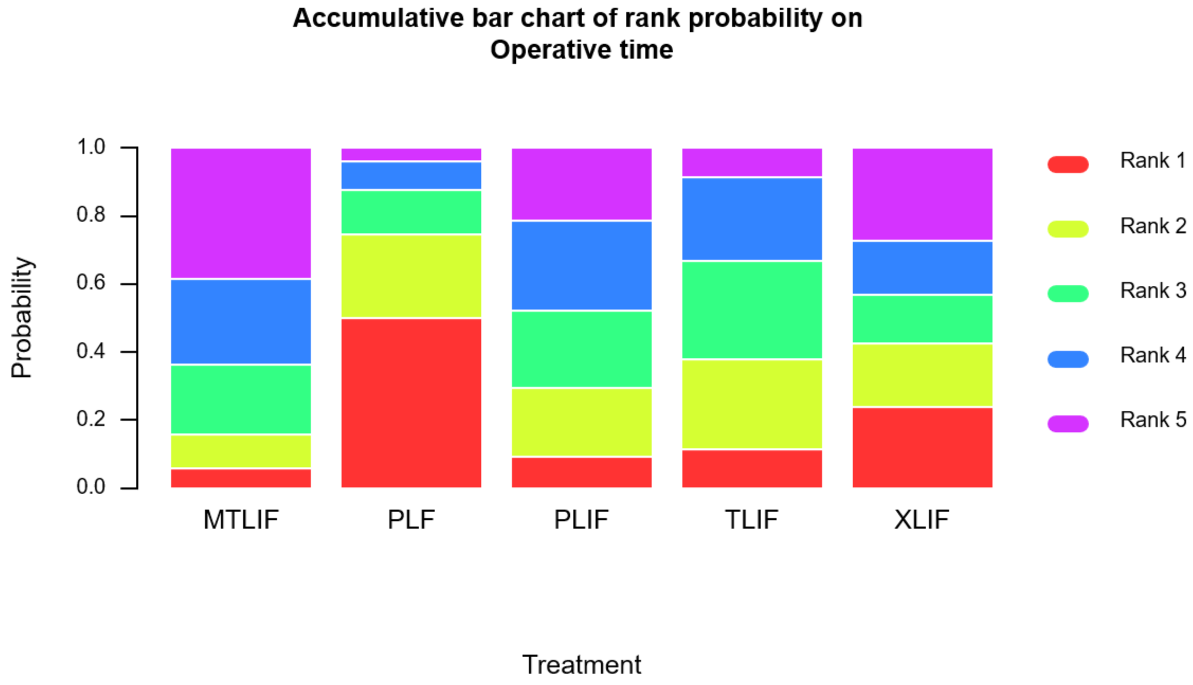

**Figure S29** Series of line charts of cumulative probability of every surgical procedure on possible rank for operative time. MTLIF, minimally invasive transforaminal interbody fusion; PLF, posterolateral fusion; PLIF, posterior lumbar interbody fusion; TLIF, transforaminal interbody fusion; XLIF, extreme lateral interbody fusion.

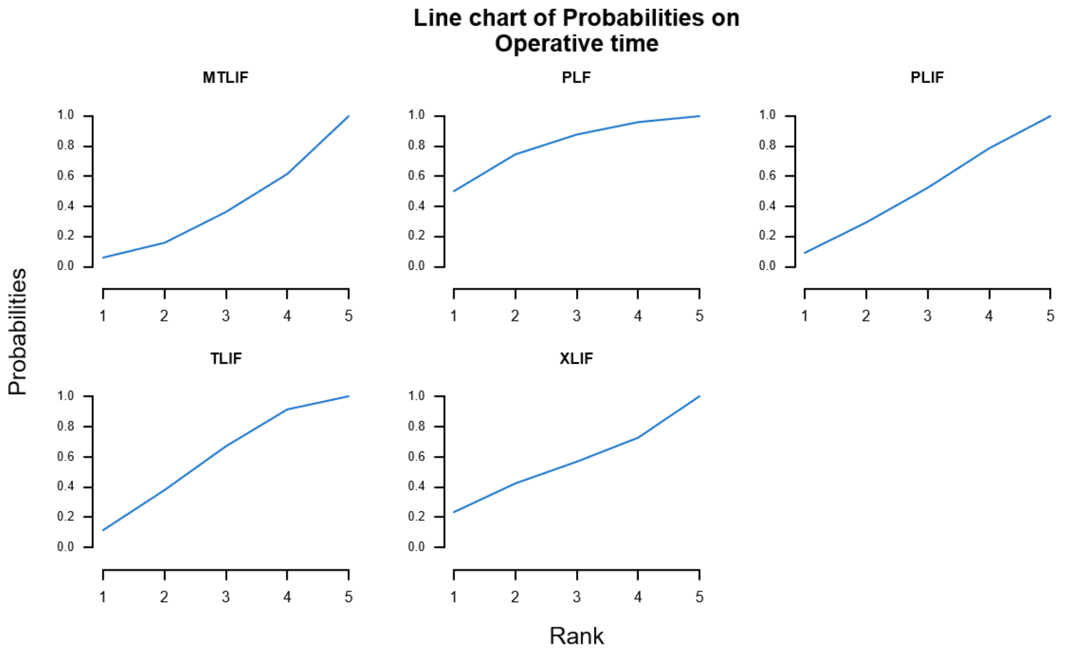


**Figure S30** Multi-line plot of cumulative probability of every surgical procedure on possible rank for operative time. MTLIF, minimally invasive transforaminal interbody fusion; PLF, posterolateral fusion; PLIF, posterior lumbar interbody fusion; TLIF, transforaminal interbody fusion; XLIF, extreme lateral interbody fusion.

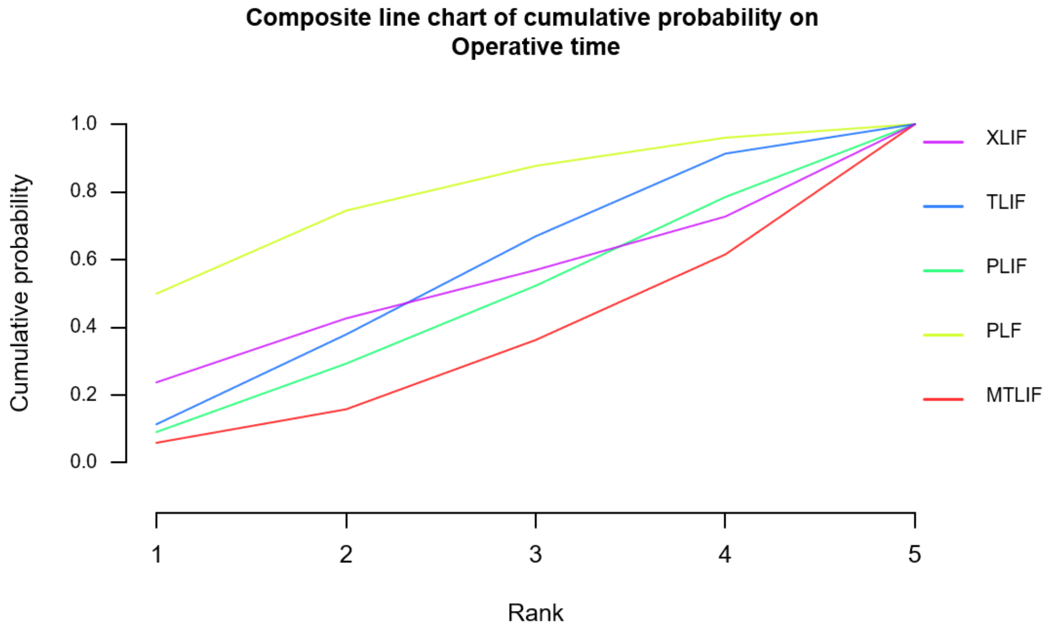

**Figure S31** Simple bar charts of global metrics of treatment rankings on operative time using (A) P-score and (B) SUCRA. MTLIF, minimally invasive transforaminal interbody fusion; PLF, posterolateral fusion; PLIF, posterior lumbar interbody fusion; TLIF, transforaminal interbody fusion; XLIF, extreme lateral interbody fusion.

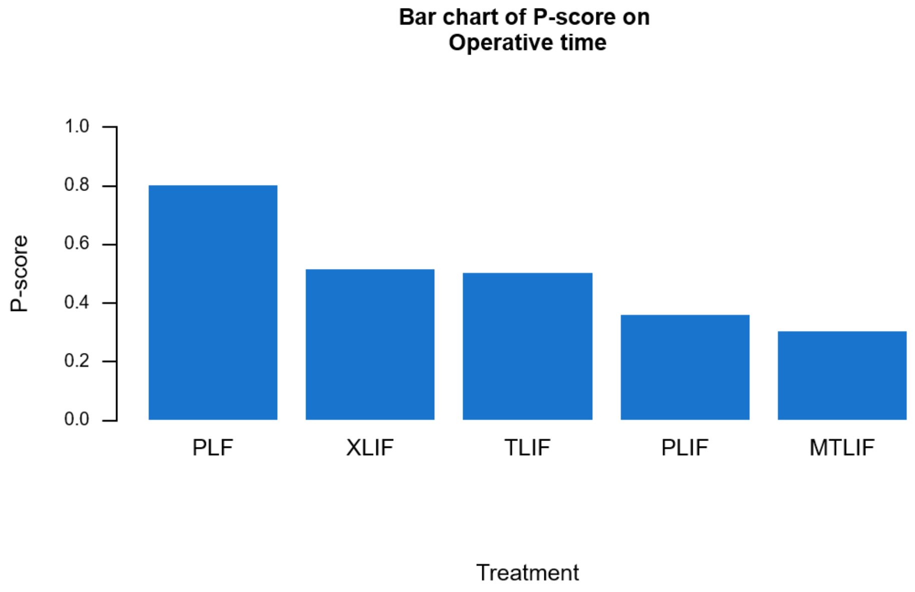


(A)

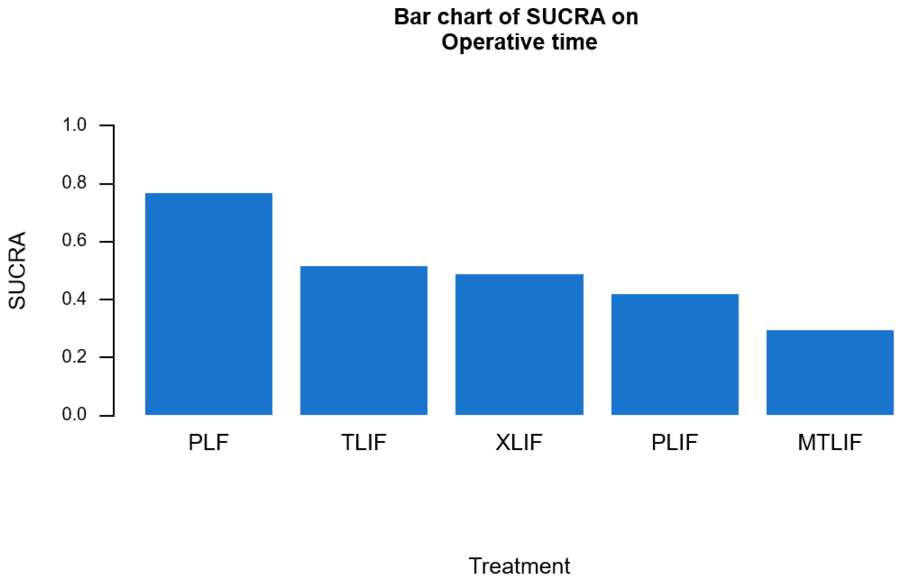


(B)

**Figure S32** Simple line charts of global metrics of treatment rankings on operative time using (A) P-score and (B) SUCRA. MTLIF, minimally invasive transforaminal interbody fusion; PLF, posterolateral fusion; PLIF, posterior lumbar interbody fusion; TLIF, transforaminal interbody fusion; XLIF, extreme lateral interbody fusion.

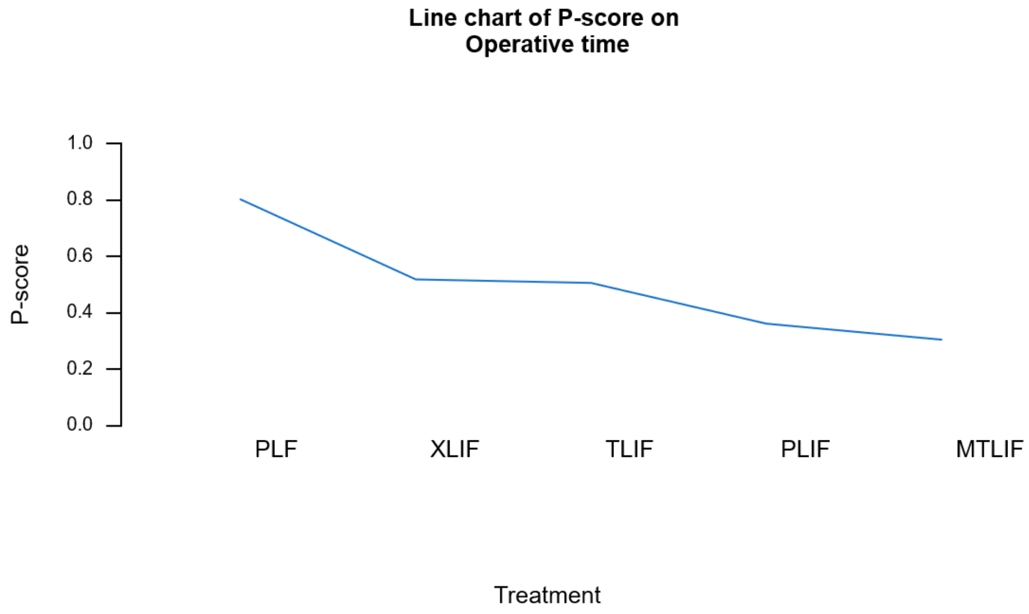


(A)

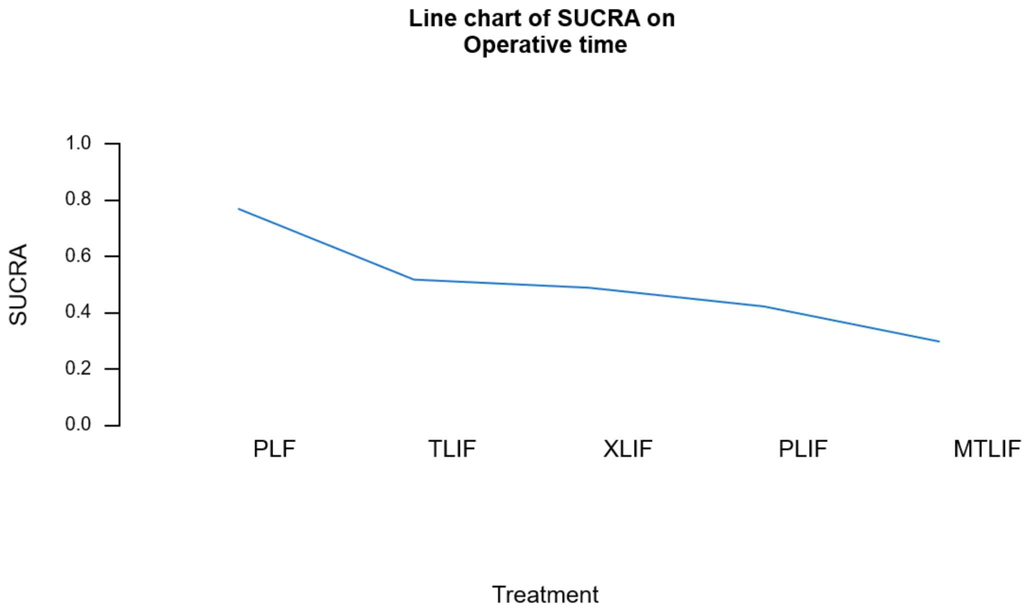


(B)

**Figure S33** Heat plots of global metrics of treatment rankings on operative time using (A) P-score and (B) SUCRA. MTLIF, minimally invasive transforaminal interbody fusion; PLF, posterolateral fusion; PLIF, posterior lumbar interbody fusion; TLIF, transforaminal interbody fusion; XLIF, extreme lateral interbody fusion.

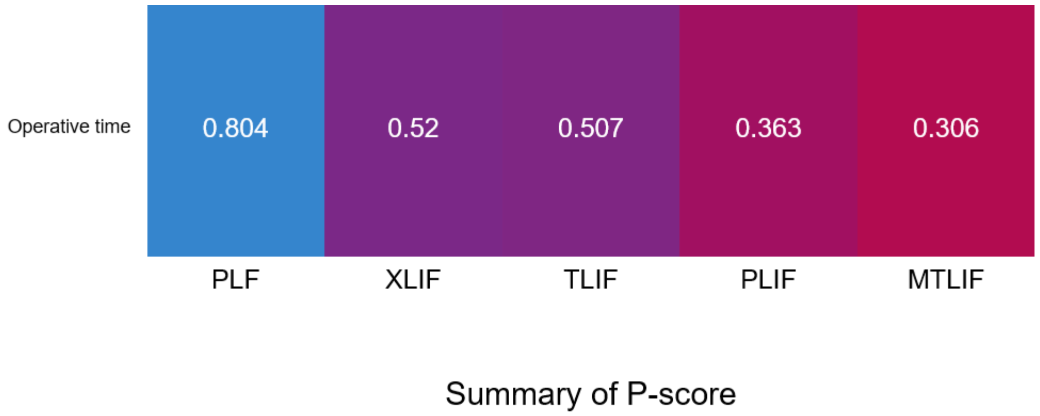


(A)

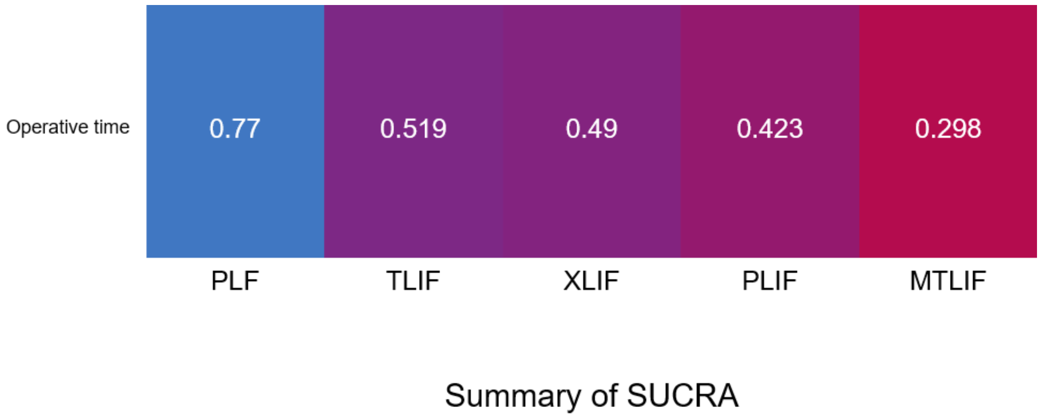


(B)

**Figure S34** Spie plot of network meta-analysis on four outcomes using (A) P-score and (B) SUCRA. MTLIF, minimally invasive transforaminal interbody fusion; PLF, posterolateral fusion; PLIF, posterior lumbar interbody fusion; TLIF, transforaminal interbody fusion; XLIF, extreme lateral interbody fusion.

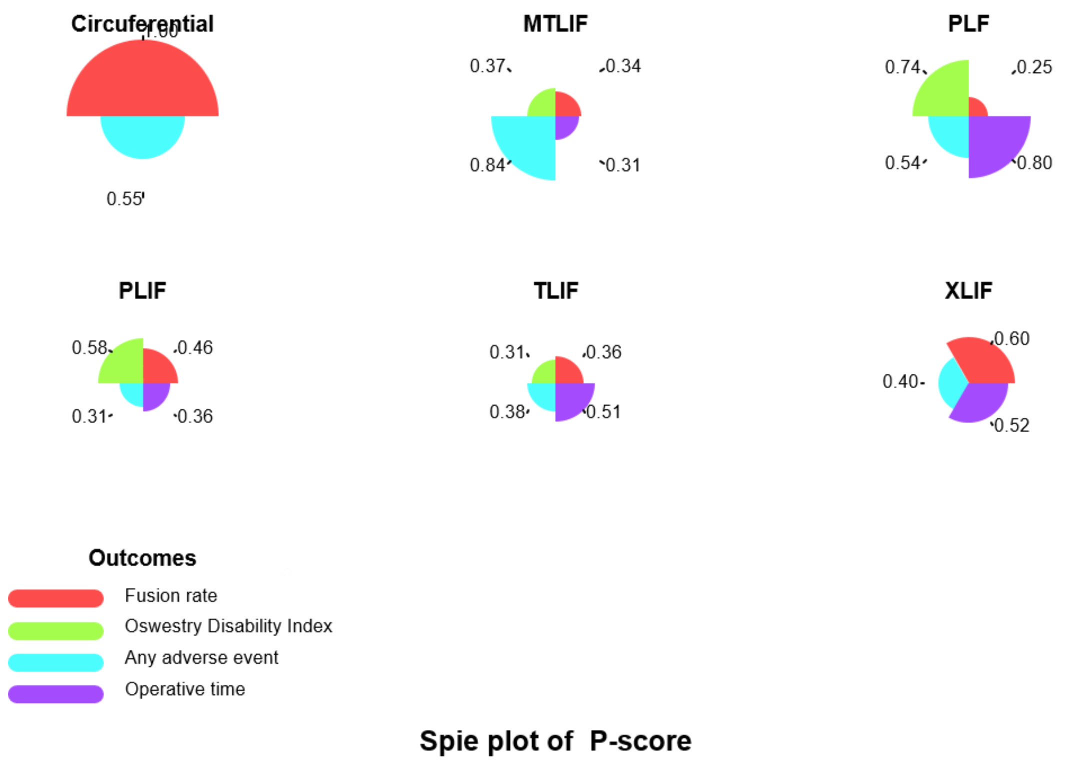


(A)

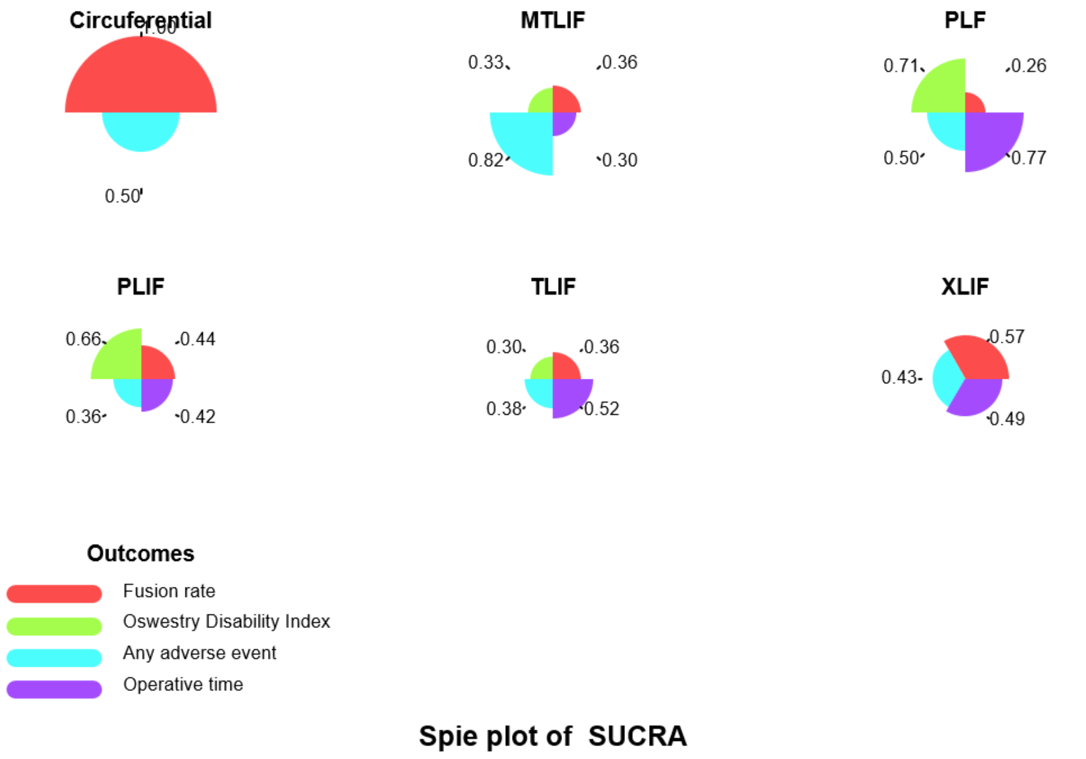


(B)

**Figure S35** Rank-heat plot of network meta-analysis on four outcomes using SUCRA. MTLIF, minimally invasive transforaminal interbody fusion; PLF, posterolateral fusion; PLIF, posterior lumbar interbody fusion; TLIF, transforaminal interbody fusion; XLIF, extreme lateral interbody fusion.

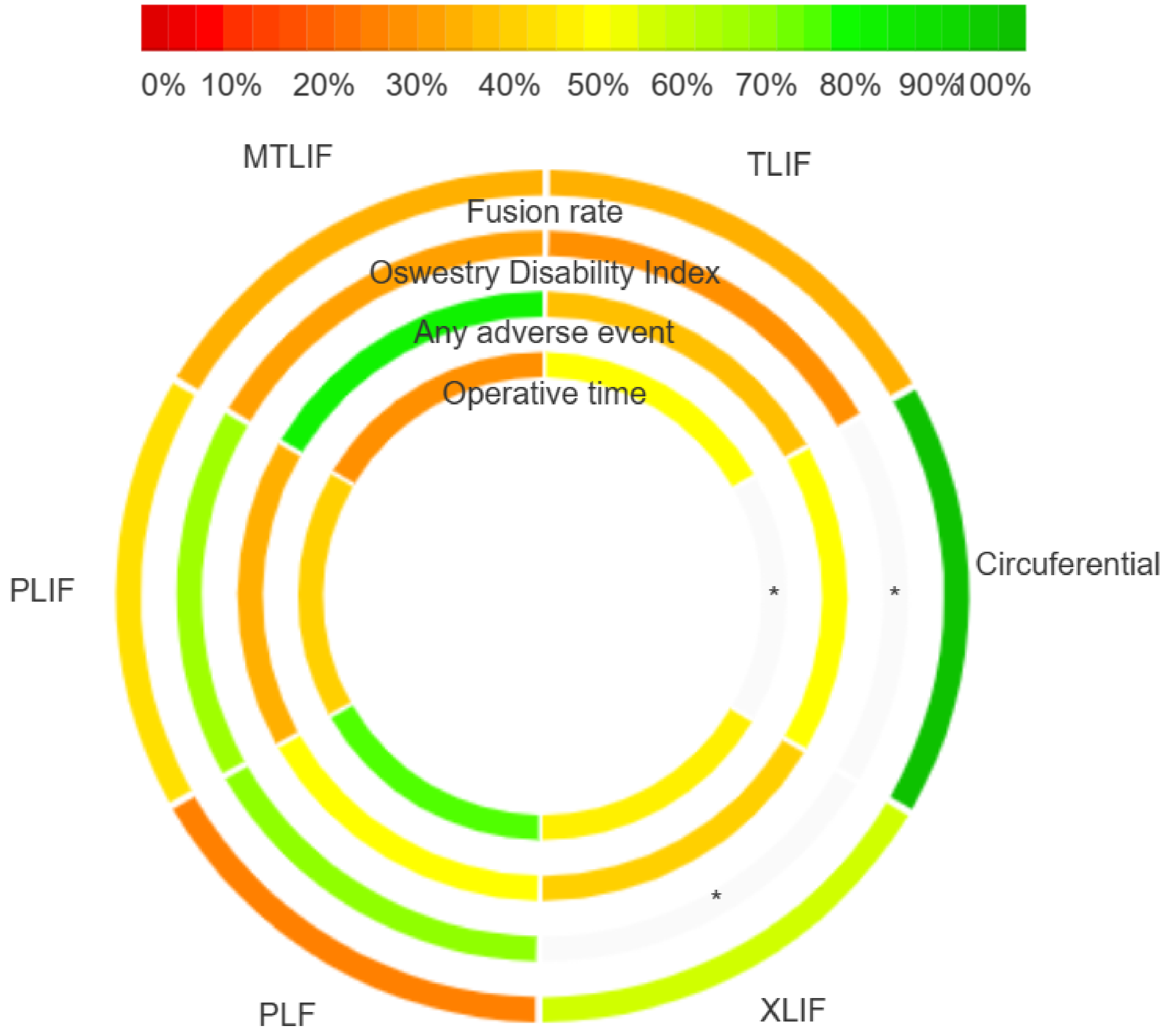

**Figure S36** Scatter plot of global metrics of treatment rankings by (A) P-scores between fusion rate and adverse event, (B) SCURA between fusion rate and adverse event. MTLIF, minimally invasive transforaminal interbody fusion; PLF, posterolateral fusion; PLIF, posterior lumbar interbody fusion; TLIF, transforaminal interbody fusion; XLIF, extreme lateral interbody fusion.

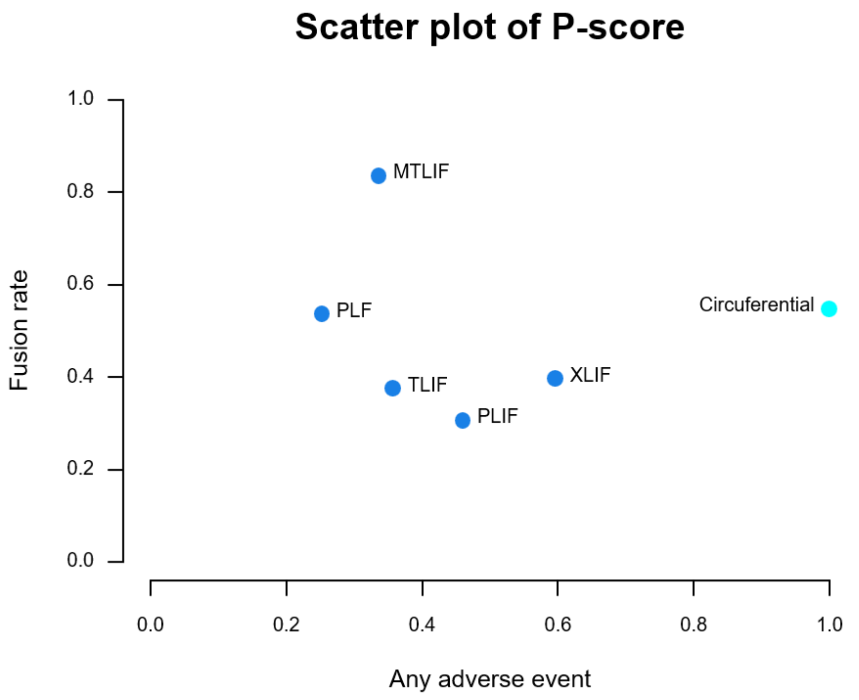


(A)

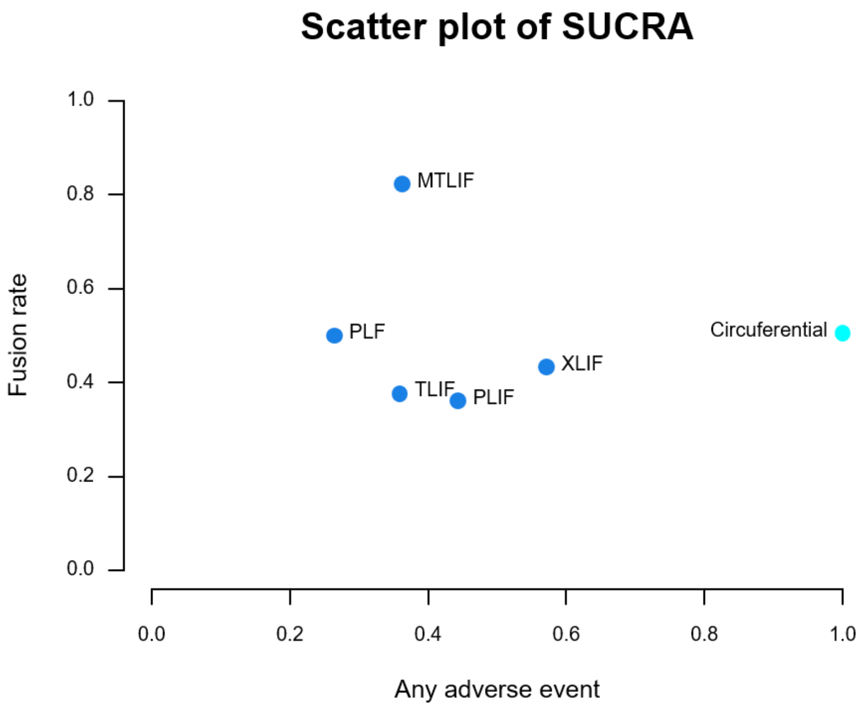


(B)

**Figure S37** Colorblind-friendly beading plot of global metrics of treatment rankings by SCURA. MTLIF, minimally invasive transforaminal interbody fusion; PLF, posterolateral fusion; PLIF, posterior lumbar interbody fusion; TLIF, transforaminal interbody fusion; XLIF, extreme lateral interbody fusion.

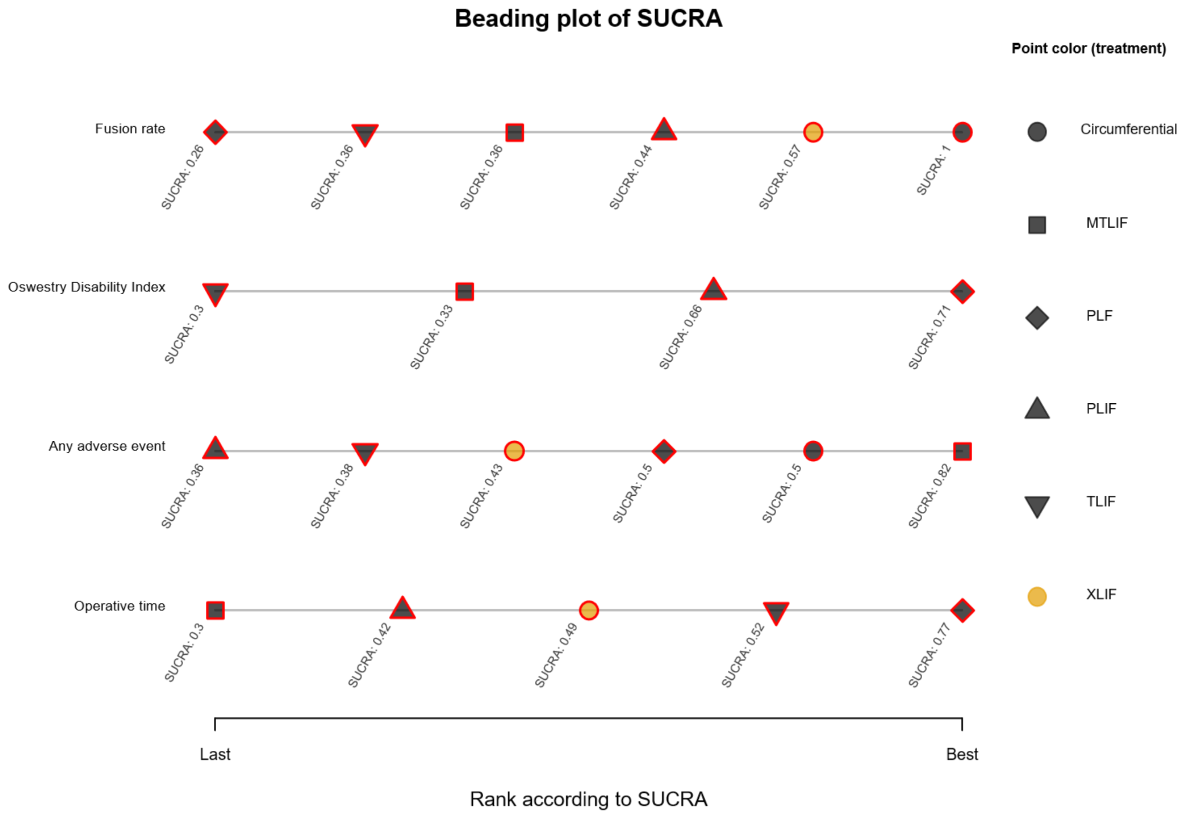


Note: red border refers to high risk of bias

**Table S1** Further information for beading plot

| Outcome / Treatment | Relative effect | Majority of risk of bias[a] |
|---|---|---|
| **Fusion rate[b]** | | |
| Circumferential | 2.100 | High |
| MTLIF | 0.990 | High |
| PLF | 0.975 | High |
| PLIF | 1.000 | High |
| TLIF | 0.990 | High |
| XLIF | 1.043 | High |
| **Oswestry Disability Index[c]** | | |
| MTLIF | 1.750 | High |
| PLF | -0.301 | High |
| PLIF | 0.000 | High |
| TLIF | 1.900 | High |
| **Any adverse event[b]** | | |
| Circumferential | 0.623 | High |
| MTLIF | 0.184 | High |
| PLF | 0.686 | High |
| PLIF | 1.000 | High |
| TLIF | 0.919 | High |
| XLIF | 0.840 | High |
| **Operative time[c]** | | |
| MTLIF | 13.400 | High |
| PLF | -49.429 | High |
| PLIF | 0.000 | High |
| TLIF | -11.600 | High |
| XLIF | -11.600 | High |

[a] risk of bias is obtained based on Kang YN, Ho YW, Chu W, Chou WS, Cheng SH. Effects and Safety of Lumbar Fusion Techniques in Lumbar Spondylolisthesis: A Network Meta-Analysis of Randomized Controlled Trials. Global Spine J. 2022;12(3):493-502. doi:10.1177/2192568221997804. [b] risk ratio. [c] mean difference. MTLIF, minimally invasive transforaminal interbody fusion; PLF, posterolateral fusion; PLIF, posterior lumbar interbody fusion; TLIF, transforaminal interbody fusion; XLIF, extreme lateral interbody fusion.

**File S1** Steps and R code for generating beading plot.

```
# Dataset:
# dataFR is data set of fusion rate (dichotomous data)
# dataODI is data set of Oswestry Disability Index score (continuous data)
# dataAE is data set of adverse event (dichotomous data)
# dataOPT is data set of operative time (continuous data)
#
# Note: all the data had been calculated as contrast-level in terms of treatment effect (TE)
# with standard error of treatment effect (seTE)

# Call library “netmeta”
library(netmeta)

# Call library “rankinma”
library(rankinma)

# Conduct network meta-analysis of fusion rate
nmaFR <- netmeta(TE = TE, seTE = seTE,
                 treat1 = treat1, treat2 = treat2,
                 studlab = paste(dataFR $studlab),
                 data = dataFR,
                 reference.group = “PLIF”,
                 sm = “RR”,
                 comb.fixed = FALSE, comb.random = TRUE,
                 method.tau = “DL”,
                 details.chkmultiarm = TRUE, sep.trts = “ vs ”)

# Conduct network meta-analysis of Oswestry Disability Index score
nmaODI <- netmeta(TE = TE, seTE = seTE,
                  treat1 = treat1, treat2 = treat2,
                  studlab = paste(dataODI$studlab),
                  data = dataODI,
                  reference.group = “PLIF”,
                  sm = “MD”,
                  comb.fixed = FALSE, comb.random = TRUE,
                  method.tau = “DL”,
                  details.chkmultiarm = TRUE, sep.trts = “ vs ”)

# Conduct network meta-analysis of adverse event
nmaAE <- netmeta(TE = TE, seTE = seTE,
                 treat1 = treat1, treat2 = treat2,
                 studlab = paste(dataAE$studlab),
                 data = dataAE,
                 reference.group = “PLIF”,
                 sm = “RR”,
                 comb.fixed = FALSE, comb.random = TRUE,
                 method.tau = “DL”,
                 details.chkmultiarm = TRUE, sep.trts = “ vs ”)

# Conduct network meta-analysis of operative time
nmaOPT <- netmeta(TE = TE, seTE = seTE,
                  treat1 = treat1, treat2 = treat2,
                  studlab = paste(dataOPT$studlab),
                  data = dataOPT,
                  reference.group = “PLIF”,
                  sm = “MD”,
                  comb.fixed = FALSE, comb.random = TRUE,
                  method.tau = “DL”,
                  details.chkmultiarm = TRUE, sep.trts = “ vs ”)
```

```
# Get matric P-score from output object of fusion rate
pscoreFR <- GetMetrics(nmaFR,
                       outcome = “Fusion rate”,
                       metrics = “P-score”,
                       prefer = “large”)

# Get matric from outcome of Oswestry Disability Index
pscoreODI <- GetMetrics(nmaODI,
                        outcome = “Oswestry Disability Index”,
                        metrics = “P-score”,
                        prefer = “small”)

# Get matric from outcome of adverse event
pscoreAE <- GetMetrics(nmaAE,
                       outcome = “Any adverse event”,
                       metrics = “P-score”,
                       prefer = “small”)

# Get matric from outcome of operative time
pscoreOPT <- GetMetrics(nmaOPT,
                        outcome = “Operative time”,
                        metrics = “P-score”,
                        prefer = “small”)

# Merge data of P-scores among four outcomes
dataPscore <- rbind(pscoreFR, pscoreODI, pscoreAE, pscoreOPT)

# Prepare data for drawing beading plot
dataPscore <- SetMetrics(dataPscore,
                         outcome = outcome,
                         tx = tx,
                         metrics = P.score,
                         metrics.name = “P-score”)

# Generate beading plot of four outcomes by P-score
PlotBeads(dataPscore,
          szFntY = 0.7,
          scaleX = “Numeric”,
          txtValue = “Effects”,
          rotateTxt = 60,
          szFntTxt = 0.4)


# Get matric SUCRA from output object of fusion rate
sucraFR <- GetMetrics(nmaFR,
                      outcome = “Fusion rate”,
                      metrics = “SUCRA”,
                      prefer = “large”)

# Get SUCRA from outcome of Oswestry Disability Index
sucraODI <- GetMetrics(nmaODI,
                       outcome = “Oswestry Disability Index”,
                       metrics = “SUCRA”,
                       prefer = “small”)

# Get SUCRA from outcome of adverse event
sucraAE <- GetMetrics(nmaAE,
                      outcome = “Any adverse event”,
                      metrics = “SUCRA”,
                      prefer = “small”)
```

```
# Get SUCRA from outcome of operative time
sucraOPT <- GetMetrics(nmaOPT,
                       outcome = “Operative time”,
                       metrics = “SUCRA”,
                       prefer = “small”)

# Merge data of SUCRA among four outcomes
dataSUCRA <- rbind(sucraFR, sucraODI, sucraAE, sucraOPT)

# Prepare data for drawing beading plot
dataSUCRA <- SetMetrics(dataSUCRA,
                        outcome = outcome,
                        tx = tx,
                        metrics = SUCRA,
                        metrics.name = “SUCRA”)

# Generate beading plot of four outcomes by SUCRA
PlotBeads(dataSUCRA,
          szFntY = 0.7,
          scaleX = “Rank”,
          txtValue = “Metrics”,
          rotateTxt = 60,
          szFntTxt = 0.4)
```
